# Supplementary material for: JAK inhibition decreases the autoimmune burden in Down syndrome
Source: eLife. 2024 Dec 31;13:RP99323. doi: 10.7554/eLife.99323 (PMC11687933; doi:10.7554/eLife.99323)
Supplement: Reporting standard 1. [file elife-99323-repstand1.pdf]

COMIRB Protocol
Colorado Multiple Institutional Review Board
Campus Box: F-490 **Telephone:** 303-724-1055 **Fax:** 303-724-0990

Protocol #: 19-1362
Project Title: Safety and efficacy of tofacitinib for immune skin conditions in Down syndrome
Principal Investigator: Joaquin Espinosa, PhD
Co-PIs: David Norris, MD; Emily Gurnee, MD; Angela Rachubinski, PhD

Document: COMIRB Protocol
Version Date: 08.16.2023

I. Hypotheses and Specific Aims:

The project described here is a phase II, proof-of-concept, open-label trial that will evaluate the safety and efficacy of a 16-week treatment with Tofacitinib in people with Down syndrome (DS) and an autoimmune and/or autoinflammatory skin condition, collectively referred hereto as 'immune skin conditions.' The driving hypothesis for this project is that hyperactivation of interferon (IFN) signaling leads to a myriad of immune-driven diseases and immunological phenotypes in people with Down syndrome, and that pharmacological inhibition of IFN signaling with Tofacitinib could have multidimensional therapeutic benefits in this population.

<u>Our specific aims are:</u>

1. To define the safety profile of JAK inhibition in people with DS,
2. To determine the impact of JAK inhibition on the immune dysregulation caused by trisomy 21,
3. To define the impact of JAK inhibition on immune skin conditions in DS, and
4. To characterize the impact of JAK inhibition on cognition and quality of life in DS.

<u>Accordingly, our hypotheses are:</u>

1. Treatment with Tofacitinib for 16 weeks in people with DS will not result in a higher frequency of serious adverse events definitely related to treatment than reported in the typical population.
2. Treatment with Tofacitinib for 16 weeks in people with DS will result in statistically significant reduction of IFN transcriptional scores.
3. Treatment with Tofacitinib for 16 weeks in people with DS will result in statistically significant reduction of one or more additional markers of immune dysregulation, including a) circulating levels of inflammatory cytokines, b) levels of neurotoxic metabolites in the IFN-inducible kynurenine pathway, and c) levels of autoantibodies.
4. Treatment with Tofacitinib for 16 weeks in people with DS will result in statistically significant percentage improvement in one or more measures of skin pathology, including a) Investigator's Global Assessment, b) Dermatology Life Quality Index, and c) disease-specific scoring metrics.
5. Treatment with Tofacitinib for 16 weeks in people with DS will result in statistically significant improvement in quality of life measured by one or more NIH PROMIS tools.
6. Treatment with Tofacitinib for 16 weeks in people with DS will result in statistically significant improvement in one or more measures of cognitive function.

II. Background and Significance:

Trisomy 21 (T21) is the most common human chromosomal disorder, occurring in ~1/700 live births, leading to the condition known as Down syndrome (DS) (1, 2). A remarkable observation enabled by the significant increase in the life expectancy of people with DS is that T21 causes a novel disease spectrum, protecting these individuals from some diseases, such as most solid tissue tumors (3, 4), while strongly predisposing them to others, such as Alzheimer's disease (5). Importantly, people with DS have an unequivocally high

prevalence of widespread immune dysregulation. In fact, they are likely to be the largest human population with a genetic predisposition to multi-organ autoimmunity. Multiple conditions driven by autoantibodies and autoreactive T cells show significantly elevated incidence among individuals with T21, including autoimmune thyroid disease (AITD) (6-12), celiac disease (13-19), and type 1 diabetes (20-22). Importantly, and directly related to the clinical outcomes to be evaluated in this trial, many immune skin conditions are also more prevalent in T21, including atopic dermatitis (AD), alopecia areata (AA), hidradenitis suppurativa (HS), psoriasis, and vitiligo (23-26).

Several lines of evidence support the hypothesis **that increased IFN signaling is the major driver of immune dysregulation in DS, and that pharmacological inhibition of IFN signaling could have broad therapeutic impacts in this population.** Data shown in *Preliminary Studies* below demonstrate that: 1) T21 causes consistent upregulation of the IFN transcriptional response in multiple cell types, including key immune cell types involved in control of autoimmunity (*Figure 3*) (27); 2) T21 causes changes in the circulating proteome indicative of chronic autoinflammation (28), including elevated levels of potent cytokines with mechanistic ties to IFN signaling and with established roles in human pathology (*Figure 4*); and 3) T21 causes dysregulation of tryptophan catabolism toward production of neurotoxic metabolites in the IFN-inducible kynurenine pathway (KP) (*Figure 5*) (29). Importantly, these results could be explained by the mere fact that four of the six IFN receptors (IFNRs) are encoded on chr21. Despite the wealth of data pointing to hyperactive IFN signaling as a driver of immune dysregulation in DS, the contribution of IFN signaling to DS phenotypes and associated co-morbidities has not been defined. Therefore, we propose here to test this hypothesis by completing a proof-of-concept clinical trial with multiple synergistic endpoints using a pharmacological inhibitor of IFN signaling.

A number of therapeutic strategies have been developed to inhibit IFN signaling, including anti-IFNR antibodies (30), decoy IFNRs (31-33), IFN 'vaccines' (34), and small molecule inhibitors of the JAK kinases (35-37). Among these, JAK inhibitors have been the most successful, and four JAK inhibitors have been approved by the FDA: Tofacitinib (JAK1/3, approved for rheumatoid arthritis, psoriatic arthritis, ulcerative colitis, and polyarticular juvenile idiopathic arthritis), Baricitinib (JAK1/2, for rheumatoid arthritis), Ruxolitinib (JAK1/2, for myelofibrosis, polycythemia vera, and acute graft-versus-host disease), and Upadacitinib (JAK1, for rheumatoid arthritis) (37). Multiple clinical trials have also tested JAK inhibitors for immune skin conditions with very positive results (38-47). Furthermore, in 2018 alone, the FDA granted Fast Track and Breakthrough Therapy designations to four different early-stage JAK inhibitors to treat AA and AD (48-51). However, the exclusion criteria for these trials prevent the participation of people with DS, as they pre-emptively exclude participants with a history of heart malfunction, myeloproliferative disorders, hearing issues, hypothyroidism, etc., and often require verbal or written assessments that are difficult for some people with DS to complete. Therefore, a clinical trial designed exclusively for individuals with DS is amply justified.

We propose here to utilize Tofacitinib, an FDA-approved drug known to block IFN signaling and several accompanying inflammatory pathways, to reduce IFN signaling in DS and to measure its effects via multidimensional endpoints. Given the number of studies in the typical population evaluating JAK inhibitors, and our own case studies using Tofacitinib in DS (see *Preliminary Studies* below) for immune skin conditions, we expect to see improvement in skin pathology in people with DS. In addition, we hypothesize that inhibition of chronically active IFN signaling in DS would attenuate core drivers of immune dysregulation, leading to improvements in other immune diseases and various phenotypical traits of DS potentially driven by inflammation, such as cognitive deficits. We will test these hypotheses using a battery of immune and molecular phenotyping assays, as well as cognitive testing and quality of life measures.

III. Preliminary Studies/Progress Report:

Down syndrome and co-occurring immune conditions.

In 2016, we established The Crnic Institute's Human Trisome Project (HTP, COMIRB 15-2170, www.trisome.org) to investigate mechanisms driving the different disease spectrum in DS. As of July 2019, we had enrolled 675 participants, 449 of them with DS, including 279 adults (>18 years). Analysis of

participant surveys and medical records reveals that ~70% of adults with T21 have been diagnosed with at least one immune disorder (*Figure 1A*). Similar to what has been observed in the typical population, autoimmune conditions tend to cluster in DS, with ~27% of individuals being diagnosed with two or more such conditions (*Figure 1A-B*). AITD is the most common immune disorder in our cohort (~56% of adults), in agreement with reports showing anti-thyroid antibodies being present in ~60% of adults with DS (9-11, 52). Surprisingly, immune skin disorders are the second most common diagnosis among immune disorders, occurring in ~31% of participants (*Figure 1C*). The most prevalent immune skin disorder is dermatitis (mostly atopic), followed by HS, AA, psoriasis, and vitiligo (*Figure 1B*). Upon closer analysis of medical history data, we noted that many individuals self-report issues like “rough” skin, and that some general practitioners provide non-specific skin diagnoses like “boils” or “folliculitis.” However, in many cases a dermatologist was never consulted for a proper assessment and diagnosis. Thus, we anticipate that many more individuals in our cohort may have undiagnosed immune skin conditions. Finally, also of high prevalence in our cohort is celiac disease, occurring in ~9% of participants (*Figure 1C*). In addition to these obvious signs of systemic immune dysregulation, people with DS display strong neuroinflammation from an early age, which could contribute to a wide range of neurological conditions more prevalent in DS, such as autism and epilepsy (53-56), while also potentiating the effects of triplication of the amyloid precursor protein (APP) gene in the development of Alzheimer’s disease (57-64).

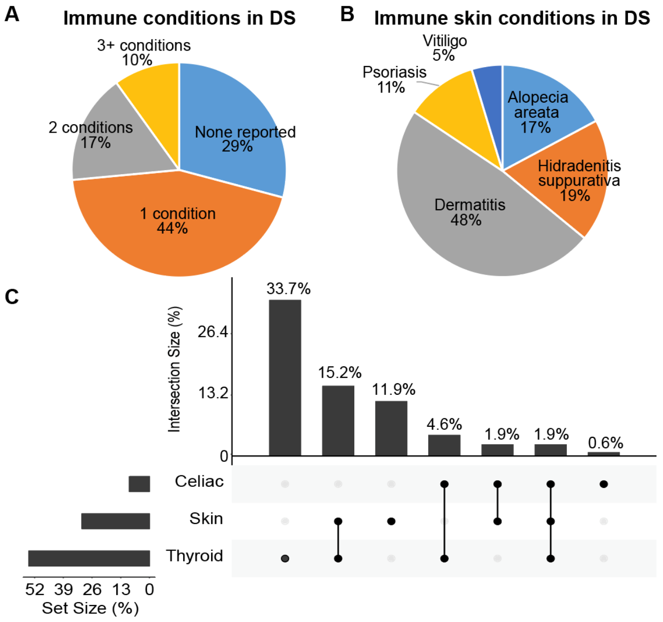

Figure 1. Individuals with Down syndrome are predisposed to diverse immune conditions. A. Pie chart showing prevalence of immune disorders in adults with DS enrolled in the Human Trisome Project. **B.** Pie chart showing types of immune skin conditions reported. **C**. Upset plot showing overlap of immune conditions in adults with DS.

Our HTP study currently includes two participants with DS who are taking Tofacitinib for AA who have had remarkable recovery of hair loss. The first participant developed an ophiasis pattern of AA when she was 6 years old. This AA pattern, affecting the lower occipital area of the scalp, is most common in individuals with DS (23, 24), and is typically very resistant to treatment (65). We have collected more than a decade of extensive clinical history from both her primary care provider and her dermatologist. At the age of 16, the AA began to spread to additional areas of the scalp, to which topical steroid and monthly injections of corticosteroids were administered at up to 12 sites in the scalp for two years to little effect. Her dermatologist, who became aware of the results of the clinical trials of JAK inhibitors for AA in typical people (35, 38-45, 47, 66-68), then requested Tofacitinib through Pfizer’s Compassionate Access program. Within one month of taking 5mg BID, fine hair re-growth was evident, at two months hair growth was dramatic, and at 3 months hair re-growth was almost complete (*Figure 2A*). The participant continues to cycle on and off the drug at approximate 3-month intervals of BID, SID, and washout periods. During the 4th cycle, at age 23, the participant experienced an almost complete re-growth of hair in the area affected by ophiasis AA for the first time in 15 years. After six years of taking Tofacitinib, the participant has not reported any undesired side effects, with consistently normal blood test results. More recently, Dr. Norris from our study team began treatment with Tofacitinib of a 15-year-old male with DS and AA. This individual reported having thick hair prior to age 13, when AA developed with intermittent patchy hair loss over the entirety of the scalp. At age 15, AA significantly worsened over the course of six

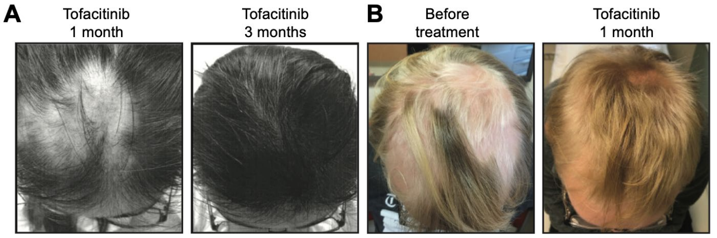

Figure 2. Individuals with DS and AA treated with the JAK inhibitor Tofacitinib. Pictures showing rapid hair recovery during Tofacitinib treatment for 23-year-old female (A) and 15-year-old male (B).

months to >50% hair loss (*Figure 2B*). The participant then began 5mg BID Tofacitinib treatment and topical clobetasol external solution (0.05%) once daily, which resulted in fluffy, down-like hair regrowth after one week. After two months on Tofacitinib, the participant showed complete regrowth of hair with no small patches of hair loss on the parietal, occipital, temporal, frontal or vertex areas, and subsequent growth restored the texture and color of his normal hair (*Figure 2B*), and the clobetasol solution was discontinued. Cell blood counts and comprehensive metabolic panel performed after eight weeks on Tofacitinib showed no changes from baseline bloodwork. No adverse effects have been noted and the individual remains on 5mg Tofacitinib twice daily.

<u>Molecular characterization of immune dysregulation caused by T21.</u>

In 2016, we reported the results from the most thorough characterization of the gene expression changes produced by T21 to date (27). Using a combination of RNA-seq, SOMAscan proteomics, and shRNA screening, we found that T21 consistently activates the IFN response. We reported the results of RNA-seq analyses from four different cell types with and without T21: skin fibroblasts, B cell lymphoblastoids, and freshly isolated T cells and monocytes (27). These efforts identified a genome-wide signature dominated by overexpression of IFN-stimulated genes (ISGs) downstream of Type I, II, and III IFN signaling (*Figure 3A*). Ingenuity Pathway Analysis (IPA) of the RNA-seq data predicts that this signature can be explained in large measure by activation of components of the three types of IFN signaling (*Figure 3B*). Notably, the JAK1 kinase is required for all three types of IFN signaling (*Figure 3A*), providing strong rational for selecting an inhibitor of JAK1 for use in T21. Using these data, we also determined that individuals with DS have elevated Type I "IFN scores" in T cells (*Figure 3C*). IFN scores have been developed repeatedly for other IFN-driven conditions (69-71), and consist of an composite score that integrates mRNA expression values for multiple ISGs into a single numerical value that can be used to track correlations with disease severity (71). IFN scores have also proven effective to monitor the impact of JAK inhibition in clinical trials (69). In the clinical trial proposed here, we will use IFN scores as a Primary Endpoint to assess the efficacy of Tofacitinib to attenuate IFN signaling in DS (see *Outcome Measures* below).

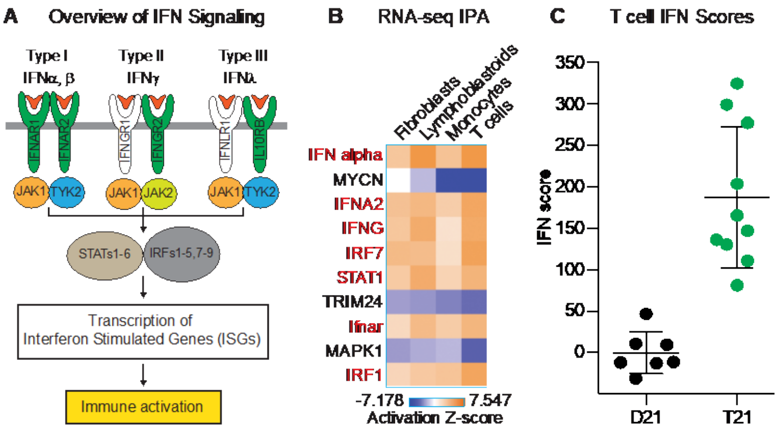

Figure 3. Trisomy 21 consistently activates the Interferon response. A. A genome-wide signature is dominated by overexpression of ISGs downstream of Type I, II, and III IFN signaling. **B.** IPA upstream regulator analysis of RNA-seq data reveals consistent activation of the IFN transcriptional response in T21 fibroblasts, lymphoblastoids, monocytes, and T cells. **C.** Example of IFN alpha activation scores driven by elevated ISG expression in T cells with T21 versus euploid controls (D21).

In 2017, we reported the results of the largest plasma proteomics study of people with DS to date (28). This study involved four cohorts totaling 263 participants, 165 of them with DS, ages six months to 43 years. Using SOMAscan technology to measure up to 3,585 proteins in plasma, we identified 299 proteins that are differentially

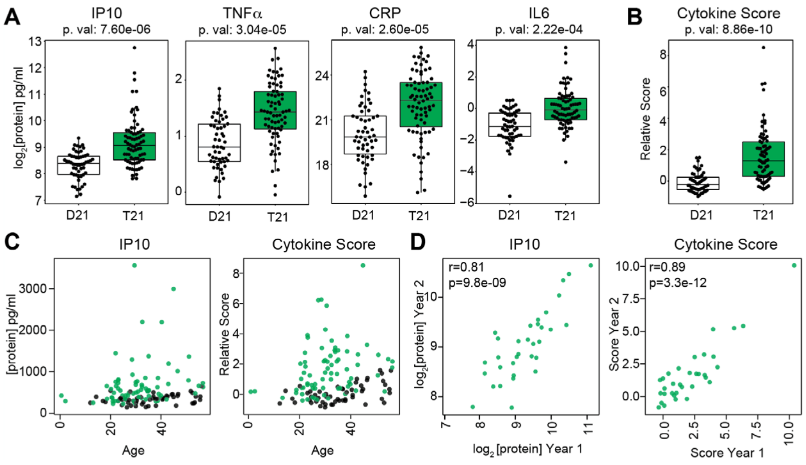

Figure 4. T21 causes changes in the circulating proteome indicative of chronic autoinflammation with links to IFN signaling. A. Box and whisker plots for elevated inflammatory cytokines in people with DS detected by MSD assays (n=54 D21 versus 75 T21). p values calculated with KS test. **B.** Cytokine score created as a composite Z score for the four cytokines in (A). **C.** Values for individual cytokines or cytokine scores do not show obvious age-dependency. **D.** Among people with DS with varying degrees of cytokine levels, their inflammatory markers are stable over a year (n= 33).

abundant in the circulation of people with DS, with ~50% of these being involved in control of the immune system (28). Recently, we expanded on these SOMAscan results by measuring a set of 55 cytokines using Meso Scale Discovery (MSD) assays in a cohort of 129 individuals (75 with T21), which revealed that people with DS have significantly elevated levels of potent inflammatory cytokines and chemokines known to act downstream of IFN signaling with known roles in immune conditions, such as IP10 (IFN-inducible protein 10), TNFα, CRP, and IL6 (*Figure 4A*). These findings enabled us to create a 'Cytokine Score', aggregating the Z-scores for these four cytokines (*Figure 4B*). We then asked whether elevation of individual cytokines or the composite cytokine score was age-dependent, which revealed no obvious correlation with age, regardless of karyotype (*Figure 4C*). Finally, we analyzed these values for the same individuals sampled one year apart, which revealed a remarkable stability in their inflammatory markers (*Figure 4D*). Thus, although inflammation is highly variable among people with DS, the degree of inflammation seems to be stable over time for a given individual, which could explain the high inter-individual variability in the phenotypic manifestation of immune conditions in DS. In the clinical trial proposed here, we will use MSD technology to analyze levels of inflammatory cytokines as another strategy to assess the efficacy of Tofacitinib to reduce immune dysregulation in DS (see *Outcome Measures* below).

Finally, we recently completed a plasma metabolomics study using mass spectrometry on 170 samples, 72 of them from individuals with DS, which revealed that T21 causes profound dysregulation of tryptophan (TRP) metabolism, leading to elevated levels of kynurenine (KYN) and its derivative quinolinic acid (QA) in people with DS (*Figure 5A-C*) (29). These results could be explained by induction of the IDO1 enzyme, a known ISG that catalyzes the rate-limiting step in this pathway (72), and which we found to be significantly overexpressed in immune cells of people with DS (*Figure 5D*). Furthermore, we found a strong positive correlation between activation of the KYN pathway and levels of inflammatory cytokines such as TNFα (*Figure 5E*). The observed upregulation of QA is a critical result, because QA is a potent neurotoxic metabolite that acts as a super-agonist of NMDA receptors, and a known driver of pathology in many neuro-degenerative diseases (73). In mouse models of DS, counteracting QA neurotoxicity with the NMDA antagonist memantine was shown to reverse cognitive defects (74-77), and memantine is currently being tested in clinical trials for cognition in DS (NCT02304302). Importantly, KYN is also known to drive neuropathology (78-84). Altogether, these results support our hypothesis that JAK inhibition could improve cognitive function in DS by decreasing IFN signaling, IDO1 expression, and KYN/QA production. In the clinical trial proposed here, we will perform targeted plasma metabolomics using these established pipelines to assess whether JAK inhibition with Tofacitinib normalizes levels of metabolites in the TRP pathway (see *Outcome Measures* below).

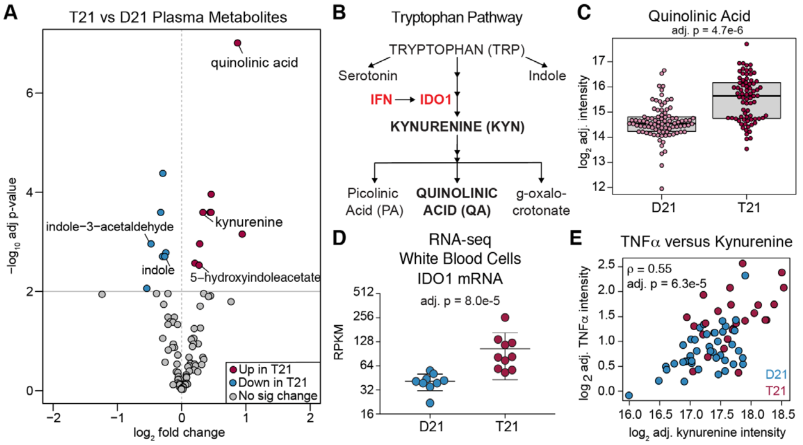

Figure 5. Trisomy 21 induces the kynurenine pathway. A. Volcano plot showing results of a plasma metabolomics experiment on 120 participants, 72 of them with T21. **B.** Overview of tryptophan catabolism pathways. **C**. Quinolinic acid (QA) is significantly elevated in plasma of people with DS. **D**. RNA-seq shows that *IDO1* is overexpressed in white blood cells from people with DS. **E.** Spearman correlation analyses revealed strong association between activation of the kynurenine pathway and circulating levels of cytokines such as TNFα measured in the same plasma

<u>Cognition in Down syndrome.</u>

All individuals with DS have some degree of cognitive impairment which most often occurs in the mild to moderate range of disability. The areas of executive function, long-term (episodic) memory, spatial processing, and processing speed, typically present more pronounced challenges relative to overall levels of delay (85, 86). Neuroanatomical features associated with DS may be linked to hyperactive IFN signaling include demyelination, atypical hippocampal presentation, and cerebellar atrophy (73, 74, 87, 88). These neuroanatomical features could obviously contribute to the neurocognitive phenotypes associated with DS (89), and some may pose an irreversible constraint on cognitive function. However, a reduction of the

neuroinflammatory phenotype of DS (90-98) may have therapeutic benefits, even in adults. Given the well-documented negative impact of hyperactive IFN signaling on brain function, from excessive synapse pruning by IFN-activated microglia to global impairments in cognitive function upon acute IFNα administration (99-106), **we hypothesize that reducing IFN signaling via JAK inhibition could improve cognition and behavior regulation in DS**. Of note, Tofacitinib was shown to cross the blood-brain barrier in mice (107). Thus, we will assess changes in selected cognition and overall quality of life metrics for individuals with DS upon JAK inhibition.

IV. Research Methods

A. Outcome Measure(s):

Name. Evaluate the safety of Tofacitinib in people with DS.
Type. Primary Endpoint.
Time Frame. Baseline through 16 weeks.
Brief Description. Adverse events (AEs) will be recorded, classified, and closely monitored by study coordinators and doctors. Based on published safety data, we calculate that we should have no more than two serious adverse events (SAE) definitely related to treatment over the course of 16-weeks in order to demonstrate comparable safety to the typical population.

Name. Change in IFN scores in the transcriptome of white blood cells.
Type. Primary Endpoint.
Time Frame. Baseline and 16 weeks.
Brief Description. Using published methods, IFN scores will be calculated using RNA-seq from whole blood RNA. A two-sided, paired Student's t-test will be used to determine the significance of any changes observed.

Name. Change in IGA.
Type. Secondary Endpoint.
Time Frame. Baseline and 16 weeks.
Brief Description. The Investigator's Global Assessment (IGA) is commonly used in clinical trials to assess overall severity of skin disease and is undiscerning of any one specific skin condition. A two-sided, paired Student's t-test will be used to determine the significance of any changes observed.

Name. Change in DLQI.
Type. Secondary Endpoint.
Time Frame. Baseline and 16 weeks.
Brief Description. The Dermatology Life Quality Index (DLQI) is a patient reported outcome commonly used in clinical trials to assess quality of life as it relates specifically to skin diseases. A two-sided, paired Student's t-test will be used to determine the significance of any changes observed.

Name. Change in EASI in participants with AD.
Type. Secondary Endpoint.
Time Frame. Baseline and 16 weeks.
Brief Description. The Eczema Area and Severity Index (EASI) is commonly used in clinical trials to assess AD. A two-sided, paired Student's t-test will be used to determine the significance of any changes observed.

Name. Change in SALT in participants with AA.
Type. Secondary Endpoint.
Time Frame. Baseline and 16 weeks.
Brief Description. The Severity of Alopecia Tool (SALT) is commonly used in clinical trials to assess AA. A two-sided, paired Student's t-test will be used to determine the significance of any changes observed.

Name. Change in MSS in participants with HS.
Type. Secondary Endpoint.
Time Frame. Baseline and 16 weeks.
Brief Description. The Modified Sartorius Score (MSS) is commonly used in clinical trials to assess HS. A two-sided, paired Student's t-test will be used to determine the significance of any changes observed.

Name. Change in PASI in participants with psoriasis.
Type. Secondary Endpoint.
Time Frame. Baseline and 16 weeks.
Brief Description. The Psoriasis Area and Severity Index (PASI) is commonly used in clinical trials to assess psoriasis. A two-sided, paired Student's t-test will be used to determine the significance of any changes observed.

Name. Change in VETI in participants with vitiligo.
Type. Secondary Endpoint.
Time Frame. Baseline and 16 weeks.
Brief Description. The Vitiligo Extent Tensity Index (VETI) is commonly used in clinical trials to assess vitiligo. A two-sided, paired Student's t-test will be used to determine the significance of any changes observed.

Name. Change in levels of inflammatory cytokines.
Type. Secondary Endpoint.
Time Frame. Baseline and 16 weeks.
Brief Description. The Meso Scale Discovery platform will be used with a custom V-PLEX kit to assess levels of multiple cytokines elevated in people with Down syndrome, including four cytokines that will be used to create a composite Cytokine Score: IP10, TNFα, CRP, IL6. A two-sided, paired Student's t-test will be used to determine the significance of any changes in the Cytokine Score observed.

Name. Change in levels of neurotoxic metabolites in the kynurenine pathway.
Type. Tertiary Endpoint.
Time Frame. Baseline and 16 weeks.
Brief Description. Using established mass spectrometry protocols, identification and quantification of kynurenine, quinolinic acid, and the kynurenine/tryptophan ratio will be performed. A two-sided, paired Student's t-test with FDR correction will be used to determine the significance of any changes observed.

Name. Change in levels of autoantibodies related to AITD.
Type. Tertiary Endpoint.
Time Frame. Baseline and 16 weeks.
Brief Description. Levels of autoantibodies associated with hypothyroidism including anti-thyroperoxidase (TPO), anti-thyroglobulin (TG), and anti-thyroid stimulating hormone receptor (TSHr), will be assessed using established clinical assays. A two-sided, paired Student's t-test with FDR correction will be used to determine the significance of any changes observed.

Name. Change in levels of celiac disease-associated autoantibodies.
Type. Tertiary Endpoint.
Time Frame. Baseline and 16 weeks.
Brief Description. Levels of autoantibodies associated celiac disease including anti-tissue transglutaminase (tTG) and anti-deamidated gliadin peptide (DGP), will be assessed using established clinical assays. A two-sided, paired Student's t-test with FDR correction will be used to determine the significance of any changes observed.

Name. Change in Leiter 3 International Performance Scale scores.
Type. Tertiary Endpoint.

Time Frame. Baseline and 16 weeks.
Brief Description. The Leiter 3 cognitive battery provides a broad non-verbal assessment of cognitive function, including executive function and spatial processing. We will employ the Sustained attention, Nonverbal Stroop Tasks, Forward and Backward Memory and Sequential Order subdomains, to assess inhibitory control, cognitive flexibility, working memory, and spatial processing respectively. An FDR-corrected Wilcoxon signed-rank test (matched pairs) will be used to determine the significance of any changes observed.

Name. Change in CANTAB scores.
Type. Tertiary Endpoint.
Time Frame. Baseline and 16 weeks.
Brief Description. The Cambridge Neuropsychological Test Automated Battery (CANTAB) assesses motor response time and working memory quickly and reliably in individuals with Down syndrome. The CANTAB Paired Associate Learning subdomain will be used to measure episodic learning and the Reaction Time Interval subdomain used to measure processing speed. An FDR-corrected, two-sided, paired Student's t-test will be used to determine the significance of any changes observed.

Name. Change in Science of Behavior Change Simple Reaction Time scores.
Type. Tertiary Endpoint.
Time Frame. Baseline and 16 weeks.
Brief Description. The Science of Behavior Change (SOBC) Simple Reaction Time (SRT) is used to assess the reaction time upon seeing a stimulus. An FDR-corrected, two-sided, paired Student's t-test will be used to determine the significance of any changes observed.

Name. Change in NEPSY II scores.
Type. Tertiary Endpoint.
Time Frame. Baseline and 16 weeks.
Brief Description. The NEuroPSYchological Assessment, Second Edition (NEPSY II) is used to assess neuropsychological development. The Visuomotor subdomain will be used to measure motor development.

Name. Change in PROMIS survey tools of quality of life.
Type. Tertiary Endpoint.
Time Frame. Baseline and 16 weeks.
Brief Description. The NIH Patient-Reported Outcomes Measurement Information System (PROMIS) tool is a rigorously tested patient reported outcome (PRO) measurement tool that uses recent advances in information technology, psychometrics, and qualitative, cognitive, and health survey research to measure PROs that have a major impact on quality-of-life across a variety of chronic diseases. The Anxiety, Depression, and Applied Cognitive General Concerns Short Forms will be used, as well as the Positive Affect Pediatric Short form, to assess general quality of life. A two-sided, paired Student's t-test will be used to determine the significance of any changes observed.

B. Description of Population to be Enrolled:

We will enroll adolescents and adults (age 12-50) with DS and one or more active immune skin conditions, including AD, AA, HS, psoriasis, and vitiligo (see *Preliminary Studies Figure 1*). Additional immune-driven skin conditions may also be included at the discretion of our dermatologists. Our specific inclusion and exclusion criteria are:

<u>Inclusion Criteria</u>

- Males or females with DS between 12 and 50 years of age who weigh at least 40 kg.

- Diagnosis of at least one active immune skin condition, including but not limited to:
  Moderate-to-severe atopic dermatitis
  Alopecia areata affecting at least 25% of the scalp
  Moderate-to-severe hidradenitis suppurativa
  Moderate-to-severe psoriasis
  Moderate-to-severe vitiligo.
- Be willing to avoid pregnancy or fathering children.
- Must present with a study partner or legal guardian who can complete, or assist with completing, study materials as appropriate.

Exclusion Criteria

- Weigh less than 40 kg.
- Pregnancy or breast feeding.
- No study partner or legal guardian.
- Vaccination with live attenuated virus within six weeks of inclusion in the study or planned during the study.
- Clinically significant chronic or active viral infection including but not limited to HIV, hepatitis, CMV, EBV, HSV.
- Severe renal impairment.
- History of malignant solid tumor cancer within five years prior to study entry or where there is current evidence of recurrent or metastatic disease.
- Poor venous access not allowing repeated blood tests or non-compliance with venipuncture requirements.
- Prior treatment with a JAK inhibitor or with an investigational agent, device, or procedure within 21 days of enrollment.
- Concomitant treatment with other immunosuppressants (e.g. corticosteroids, methotrexate) or strong CP3A4 or CYP2C19 inhibitors or inducers (e.g. ketoconazole, fluconazole).
- Known allergies, hypersensitivity, or intolerance to Tofacitinib.
- History of thrombotic disorder.
- Superficial skin infection within 2 weeks of inclusion in the study.
- History of disseminated herpes zoster, disseminated herpes simplex, or recurrent localized dermatomal herpes zoster.
- Intravenous antimicrobial therapy within 3 months of inclusion in the study.
- Oral antimicrobials within 2 weeks of inclusion in the study.
- Participants may be excluded for other unforeseen reasons at the study doctor’s discretion.
- Unable to provide assent in cases where informed consent is obtained from other authorized representative.
- Kidney transplant within the last two years
- Any history of heart attack or stroke.
- Any history of lymphoma.
- Past or current smokers.

C. Study Design and Activities

Recruitment and Pre-Screening.

Recruitment activities for the clinical trial will employ seven major resources available to the study team:

(1) *The NIH DS-Connect® Registry.* DS-Connect was created to “encourage the exchange of information about Down syndrome research, support, and care” and currently has registered over 5300 participants with Down syndrome. We will work with the DS-Connect® team to advertise the trial, identify any potential participants, and attract interested participants in the Colorado and surrounding areas, including western Kansas, northern New Mexico and Arizona, and eastern Utah.

(2) *The Crnic Institute Human Trisome Project (HTP)*. Since start of operations in 2016, our ongoing cohort study has enrolled 450+ individuals with Down syndrome (DS), 280+ of them adults, and will be a valuable and significant resource for recruitment to the trial. We will contact all adults with DS in this cohort who have agreed to be contacted about future research opportunities and who have any history of a skin condition to provide information about the new Clinical Trial and bring participants in for screening.

(3) *The Sie Center for Down Syndrome at Children's Hospital Colorado (Sie Center).* The Sie Center has provided medical care to over 2000 individuals with DS and is among the largest provider of specialized medical care for DS in the United States. While the Sie Center focuses mainly on the pediatric population, they provide care to patients up to 25 years of age. Our team already works closely with the Sie Center for recruitment to the HTP, and we will continue to leverage this relationship to identify and recruit adolescents and young adults for the Clinical Trial, ages 12-25.

(4) *The Global Down Syndrome Foundation (Global).* Global provides community support, advocacy, and education initiatives to the population with DS in the Denver-metro area and worldwide. Global is an affiliate organization of the Crnic Institute and will partner with our team to host education and recruitment events for the local DS community. Global has been instrumental in the success of the HTP and will strongly support this trial with continued efforts to engage the community and disperse our team's IRB-approved research and recruitment materials. Our team will also partner with Global to attend Down syndrome events in the greater region for recruitment purposes.

(5) *The Denver Health Down Syndrome Adult Clinic (Adult Clinic).* The Adult Clinic is a new initiative by the Global Down Syndrome Foundation to provide continuing specialized medical care to adults with DS who have aged out of the Sie Center. The clinic operates one day per week at a Denver Health clinic, located ~15 miles away from the University of Colorado Anschutz Medical Campus. One of the primary goals of this clinic is to connect adults with DS to research opportunities, and a clinical coordinator from our team will regularly be present at the clinic to share study information with patients interested in research.

(6) *The University of Colorado Hospital (UCH).* Study doctors from the Dermatology Clinic at UCH will see participants for clinical visits for this study. A larger recruitment campaign of all patients with Down syndrome seen at UCH will allow us to reach a potential base of over 1200 individuals with DS from the surrounding region. As this hospital accepts both private and most public insurance, these recruitment efforts will allow us to reach a diverse population. Aggregate reports from electronic medical records indicate that, in the past 3 years, 89 unique patients (ages 18+) with DS were seen at UCH for a complaint of AA, AD/eczema or HS/folliculitis.

(7*) Children's Hospital Colorado (CHCO) Dermatology.* The Dermatology Department at CHCO sees patients from the multi-state area, including individuals with DS. With five dermatologists on staff, including one who is a study doctor, this new relationship offers an exciting opportunity to offer clinical trial participation to patients who may benefit.

(8) Finally, we will also work with clinicians and collaborators across the country to facilitate the referrals of their patients who may be willing and able to travel and benefit from participation in this trial. We will also use advertisements in the general community and recruit at appropriate events.

Travel costs will be re-imbursed for a participant and study partner to attend to ensure that travel is not a barrier to participation. To ensure travel costs are offered equitably, the following matrix will be used based on Google reported drive times. Additional special circumstances will be considered on a case-by-case basis.

a) Up to a one hour drive: no additional compensation for personal vehicles. Applicable ride-share coverage re-imbursed upon receipt.

b) Travel time over one hour: $0.52/mile, plus lodging at hotel adjacent to the University or compensation ($150/night), if necessary. Applicable ride-share coverage reimbursed upon receipt.

c) Out-of-state/not drivable: Compensation for coach class tickets for participant and study partner per visit, plus lodging at hotel adjacent to the University or compensation ($150/night). All flight

choices must be pre-approved by the study team. Alternatively, a single round-trip ticket and accommodation for the 4-5 months of the study will be provided to participant and study partner not willing to travel multiple times over the course of the study. Applicable ride-share coverage or rental car coverage reimbursed upon receipt.

Advertising materials, such as flyers or mailers, will be distributed through each of the recruitment sources listed above, including in person, by email and/ or mailed to home address of potential participants. Advertising materials will be tailored based on the specific audience being targeted and the current enrollment needs of the study. For example, advertising materials created for distribution at a large Down syndrome conference with a variety of attendees when the study is in its early phases of recruitment and enrollment would use broad language encompassing multiple skin conditions, such as "Research to determine whether Tofacitinib is a safe and effective treatment for *skin conditions* in people with Down syndrome." By contrast, later in the recruitment phase, the team may wish to perform targeted recruitment to increase representation of participants with AA, for example. In this case, advertising materials would be created for distribution specifically by our clinician partners to their patients with AA and would use more specific language, such as "Research to determine whether Tofacitinib is a safe and effective treatment for *alopecia areata* in people with Down syndrome." These varied language options are detailed in the COMIRB Advertising Components Submission Form, where the use of brackets around broad phrases like "skin conditions" or "immune skin conditions" indicates that we may substitute this wording for one of the specific immune skin conditions being studied, like "alopecia areata," as needed to perform targeted recruitment. In all cases, one phrase from the "Study Title or other Ad Headers" section and 1-2 phrases from the "Purpose of the Research Study" section would be selected to create the advertising material.

Pre-screening phone interviews may be performed when appropriate to assist in identifying individuals who are likely to meet all eligibility criteria. Pre-screening interview questions will collect information about a person's demographics, health history, and any active skin conditions they may have, and will walk through the trial's inclusion and exclusion criteria. Participants will be advised of exclusionary medications and the required washout period, and that they should consult their doctor before stopping any medications before the screening visit. We may also ask for photographs to be sent to the study team via encrypted email or by text (participant's preference) to allow dermatologists to perform an initial assessment of skin condition severity to prevent unnecessary screening visits from participants who clearly would not meet inclusion criteria.

Our recruitment and pre-screening efforts will be intentionally broad in order to bring in individuals with DS with any *potentially* immune-driven skin condition for a screening visit. In our experience with the HTP, many individuals self-report issues like "dry" or "rough" skin, or medical records will indicate non-specific diagnoses like "boils." However, in many cases a dermatologist was never consulted for a proper diagnosis. Thus, we anticipate that some individuals will qualify for the trial without otherwise having known that they actually have AD or HS, for instance. Based on our experience in dermatology clinical trials, we estimate that between 25-50% of individuals recruited and brought in for a screening visit will end up "failing" and will not qualify for or be willing to comply with the trial criteria. We also estimate that up to 15% of participants will be lost over the course of the ~5-month study timeline due to attrition. These considerations lead us to plan to recruit 77 individuals for screening in order to enroll 47 individuals, of whom 40 will complete the study. However, to account for additional unforeseen screen fails and attrition, we are asking to screen up to 100 participants.

<u>Informed Consent, Assent, LARs, and Consent Procedures.</u>

Upon successful recruitment and/or pre-screening of a participant, an initial screening visit will be set up to obtain informed consent and assent and definitively determine study eligibility. At the screening visit, a clinical coordinator will ensure the participant's, or their guardian who is legally able to sign the consent form (e.g. Legally Authorized Representative [LAR], or Medical Durable Power of Attorney for Health Care [MDPOA-HC] holder), full understanding of the clinical trial and its research purpose, including the study objective, risks and benefits, the right to non-participation, review processes, emergency medical treatment, and the Research Subject's Bill of Rights.

The informed consent form will describe all aspects of the study including consent for genetic research and making deidentified and/or coded data publicly available in accordance with NIH requirements. As part of the consent, participants will have the option to agree to be contacted for future expanded studies. The consent forms specify that specimens will be used solely for research purposes, that the tests performed are not intended for clinical diagnostic purposes, and that the participants will not receive results from these studies.

At the time of informed consent, participants will also be given information about the HTP (COMIRB 15-2170) and will be given the option to consent to this study as well. In cases where participants agree to dual consent in both studies, extra tubes of blood will be drawn and any samples and identified data remaining after completion of the research activities described in this clinical trial protocol would be available for ongoing, complementary studies under the HTP protocol. Participants who do not choose to dual consent to the HTP can optionally allow for their deidentified data and remaining samples to be transferred to the HTP at the end of this study.

All participants must have a legal guardian or study partner present at every study visit in order to participate in the clinical trial, regardless of cognitive ability. Adolescents under the age of 18 and some adult participants with DS will be unable to consent for themselves due to lower cognitive ability. Adult participants who have existing Legal Guardian/Legally Authorized Representative/Medical Durable Power of Attorney for HealthCare paperwork or similar proxy documented in their accessible Epic record or provided will take precedence for decisional capacity. If none of these exist, decisional capacity for consent will be made by an independent clinician.

An independent witness will also be present for assent, if necessary. If an adult with DS is cognitively unable to provide consent for themselves, a guardian who is legally able to sign the consent form may consent for the participant. In this case, and in the case of adolescent participants under the age of 18, assent must also be obtained from the participant and will not be over-ridden by the guardian’s wishes. If it is determined a participant does not have capacity to provide consent or assent, they will not be enrolled in the study. When enrolling a participant with DS, every effort will be made to include trusted representatives of a participant, while respecting the personal autonomy of all participants. Although in-person assessment of decisional capacity, consent and assent is preferable to the study team, all or part of these procedures may be completed by HIPAA-compliant Zoom meeting in order to minimize face-to-face interaction time. The independent witness described above will be included in all Zoom meetings where the study participant is directly involved. If remote consent is obtained, the consent form will be emailed to the participant to print prior to the meeting or sent by mail. The study team member will observe the participant, or their guardian, sign the consent form. The wet-ink signed consent form will be returned to the study team before any study procedures take place at the Screening appointment.

Remote consent may be obtained from a participant or guardian who has the proper guardianship documents in place. Assent may also be obtained in person or from the study participant via Zoom. If the participant declines assent, no additional study procedures will occur. The study team recognizes that remote consent / video conferencing will not be appropriate for all participants. Participant’s preference for consent administration will take priority, unless the independent witness determines that a preference for remote consent / assessment is not appropriate for a given individual.

A participant’s status will be considered ‘Enrolled’ once results from screening are available and reviewed, and the participant attends the Baseline visit.

Study Visits and Activities.

The initial screening visit will include assessments to ensure each participant is in overall fair health and would not have any additional or unnecessary risks while taking Tofacitinib. These screening assessments include: skin assessment to officially diagnose any immune skin condition, complete physical exam, collection of vitals, chest radiographs, ECG, pregnancy test for females if not surgically sterile or two years post-menopause, and tuberculosis test. A blood draw will be performed for basic safety monitoring labs, which include hematology (CBC with WBC differential) and standard blood chemistry to assess hemoglobin, serum creatinine, bicarbonate, creatine phosphokinase, liver function (ALT, AST, ALP, albumin, bilirubin),

Condition	Assessment	Minimum score
Moderate-to-severe atopic dermatitis	Eczema Area and Severity Index (EASI)	≥ 16
Alopecia areata affecting at least 25% of the scalp	Severity of the Alopecia Tool (SALT)	≥ 25
Moderate-to-severe hidradenitis suppurativa	Hidradenitis suppurativa - Physician's Global Assessment (HS-PGA)	≥ 3
Moderate-to-severe Psoriasis	Psoriasis Area and Severity Index (PASI)	≥ 10
Moderate-to-severe Vitiligo	Vitiligo Extent Tensity Index (VETI)	≥ 2

Table 1. Minimum score for inclusion criteria for each skin condition.

and lipids (LDL, HDL, triglycerides, cholesterol). Blood draws are non-fasting. If a clinically relevant LDL, HDL, or triglycerides measure is obtained *after* the Baseline visit, a fasting draw will be obtained for follow-up. At the screening visit, viral surveillance for CMV, HSV1&2, and EBV will also be performed, as well as screening for HIV and hepatitis. TSH will also be drawn at screening, 8-weeks, and 16-weeks, as well as at 28 and 40 weeks for those in the Extension Arm, for those with positive thyroid autoantibodies at screening or a known hypothyroid diagnosis, to give clinical relevance to the AITD autoantibodies. See Table 1 for minimum score for inclusion criteria for each skin condition and Table 2 for list of safety labs collected. Study doctors may offer a skin numbing cream before all study related blood draws.

Informed consent and assent forms will also be obtained before or at the screening visit. After receiving results from all screening tests and confirming eligibility, a study doctor will prescribe Tofacitinib from the UCHealth or CU Anschutz Medical Center Investigational Pharmacy. We anticipate participants will return for their baseline visit within approximately four weeks of their screening visit. If the duration between Screening and Baseline visit is longer than 4 weeks, study doctors may order screening labs again at their discretion. At the baseline visit, participants will be instructed to begin administration of oral Tofacitinib at 5 mg BID the following morning. Study visits are timed at Time 0 (baseline), 2, 4, 8, 12, and 16-weeks throughout the course of treatment, as well as 2 weeks after ending treatment (18 weeks in total to complete study visits). Participants in the Extension Arm will have an additional visit at 28 weeks with the final follow-up visit at 40-week. Each study visit will occur +/- 3-days of this schedule determined from Time 0 (baseline), except for Extension Arm visits that will occur +/- 7 days of the ideal visit schedule. Missed study visits or visits that occur outside of this window will be reported as a protocol deviation/violation and will not change the original schedule. A missed study visit that was supposed to include dispense of Tofacitinib should be rescheduled, even if outside the allowed window. Study doctors will not continue the Tofacitinib prescription without current safety labs.
Each visit will entail a physical exam, collection of vital signs, recording and monitoring of adverse events (AEs, see below), and a blood draw for the same battery of safety monitoring

Figure 6. Timeline of proposed clinical activities.

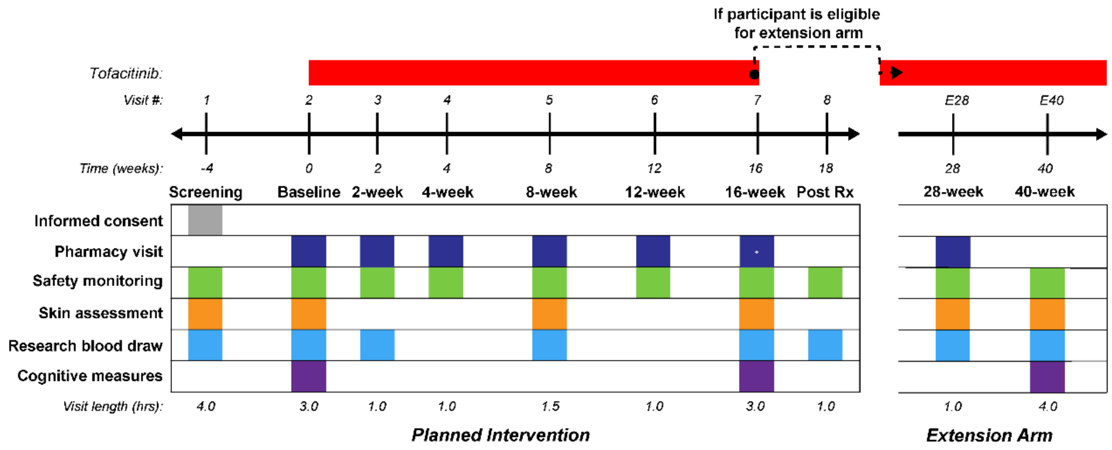

labs described in the screening procedures above. A follow-up ECG will be performed for all participants at the 4-week visit and as needed based upon concomitant medications at the study doctor's discretion. An audiogram will be performed before beginning treatment and after 15-16 weeks of treatment, to ensure Tofacitinib has not impacted hearing. A recent audiogram performed within the past 12 months for clinical reasons can be provided or accessed by the study team will be accepted, given that the appropriate elements were evaluated and no change in hearing status is anticipated. A negative Quantiferon Gold, Quantiferon Gold Plus or Quantiferon Gold HA and chest x-ray that have been obtained for clinical reasons within the past 12 months will be accepted for screening purposes if no change in TB status is anticipated. Participants will receive a tuberculosis screen at least every 12 months while in the study. Outside laboratory results will be accepted at the study doctor's discretion (*Figure 6*). Every effort will be made to have the same study doctor meet with a participant at each visit to minimize interrater bias. Skin conditions will be formally assessed at screening, baseline, 8, and 16 weeks by a dermatologist using standard and accepted metrics, such as: Investigator's Global Assessment (IGA) and Dermatology Life Quality Index (DLQI) for all skin conditions, Eczema Area and Severity Index (EASI) for AD, Severity of Alopecia Tool (SALT) for AA, Modified Sartorius Score (MSS) for HS, Psoriasis Area and Severity Index (PASI) for psoriasis, and Vitiligo Extent Tensity Index (VETI) for vitiligo. Note that the Hidradenitis Suppurativa – Physician's Global Assessment (HS-PGA) will be used to determine eligibility for participants with HS at screening and for EA qualification , while the MSS will be ongoing. Photographs will also be taken of the affected area(s) at the time of these assessments for additional validation of skin condition and visual, qualitative indicators of improvement or progression. Photographs may be taken at other safety visits to document response to treatment at the study doctor's discretion. Open-source online scoring calculators may be used to calculate skin condition severity, provided no PHI is entered. Participants in the Extension Arm will also have a formal skin assessment at 28 and 40 weeks.

Participants who have a previous diagnosis of psoriatic arthritis (PsA), or who have a suspected diagnosis of arthropathy/arthritis, who have had hand radiographs in the past 12 months will be asked to release copies using the medical record release form. Participants with PsA will also have additional observational skin assessments, including the Fingernail Assessment. The Disease Activity Score 28 (DAS28) will be collected at Visits 2, 5, 7, and 10E. Tofacitinib is an FDA-approved treatment for PsA.

Cognitive tests, including the Leiter 3 Sustained Attention, Nonverbal Stroop Tasks, Forward/Backward, and Sequential Order; the CANTAB Paired Associate Learning, Spatial Span, and Reaction Time; the NEPSY II Visuomotor; the KBIT II; the PPVT 5 Form A; SRT; and the SALT story elicitation task will be performed, and video recorded at the baseline and 16-week visits, and at 40 weeks for those in the Extension Arm. In addition, the DLQI and NIH PROMIS quality of life measures Emotional Distress-Anxiety Short Form 4a, Emotional Distress-Depression Short Form 4a, Cognitive Applied Cognitive General Concerns Short Form 4a, and Pediatric Positive Affect Short Form 4a will be administered at the baseline and 16-week visits (*Figure 6*) and 40 weeks.

Our team will collect specimens for research purposes at the screening, baseline, 2-, 8-, 16-, and 2-week post-treatment visits (*Figure 6*). These specimens include a tongue swab and additional tubes from the scheduled blood draw to support the battery of immune dysregulation analyses described in *Research Methods for Specific Aim 2* below, including DNA. Non-invasive skin tapes will be collected at baseline, 8-, and 16-weeks. All collected samples and laboratory results, such as autoantibody results, that remain unused at the end of the study or do not directly relate to the defined endpoints will be banked for future unspecified research purposes related to the goal of this study, including the possible generation of cell lines. As additional needed analyses are defined, this protocol will be amended. Research samples collected are outlined in Table 3. Participants in the Extension Arm (see below) will have the above research specimens collected at E28 and E40 as well, with the exception of skin tapes, which are only collected at E40..

Study data is collected and recorded in both electronic and paper format. Study visit data are recorded in REDCap, a HIPAA compliant online system administered locally by CU Anschutz. Study visits utilizing the CTRC are requested through the CU-Anschutz Oncore study management system, which contains study visit schedule, visit procedures, and integrates with the electronic medical record (Epic) and financial billing. Study visit procedure details are also documented in Epic.

Combined Screening and Baseline Activities.

If all screening labs and procedures are completed so that the participant is deemed eligible and can begin tofacitinib within five days of the blood draw, the laboratory values from the Screening Visit blood draw will be used for Baseline values. In this instance, an additional Baseline blood draw or formal skin assessment will not be required, except at the study doctor's discretion.

Safety Labs Collected	Version Date Implemented	Screening Visit 1	Baseline Visit 2	2-week Visit 3	4-week Visit 4	8-week Visit 5	12-week Visit 6	16-week Visit 7	2-week post Visit 8%	28-week Visit E28	40-week Visit E40
Chest X-ray	02.20.2020	X	N/A	N/A	N/A	N/A	N/A	N/A	N/A	N/A	N/A
ECG	02.20.2020	X	N/A	N/A	X	N/A	N/A	N/A	N/A	N/A	N/A
Audiogram	02.20.2020	X	N/A	N/A	N/A	N/A	N/A	X	N/A	N/A	N/A
Pregnancy Test^	04.22.2021	X	X	N/A	X	X	X	E	N/A	E	N/A
Complete Blood Count (CBC)	02.20.2020	X	X	X	X	X	X	X	X	E	E
Comprehensive Metabolic Panel	02.20.2020	X	X	X	X	X	X	X	X	E	E
Lipid Panel	02.20.2020	X	X	X	X	X	X	X	X	E	E
Creatinine Phosphokinase	02.20.2020	X	X	X	X	X	X	X	X	E	E
Quantiferon TB Gold Plus	02.20.2020	X	N/A	N/A	N/A	N/A	N/A	N/A	N/A	N/A	N/A
Virology (EBV IgG/IgM, CMV IgG/IgM, VZV IgG, HIV 1 and 2, HCV, HSV Type 1 &2 IgG, Hep B Surface Antigen/Core Antibody	02.20.2020	X	N/A	N/A	N/A	N/A	N/A	N/A	N/A	N/A	N/A
Thyroid Stimulating Hormone (TSH)	11.12.2020	X	N/A	N/A	N/A	*	N/A	X	N/A	X	X

Table 2. Schedule of safety labs. 'X'= Test performed; N/A = Not applicable. % This visit only occurs for participants who ended Tofacitinib treatment at Visit 7. E = Extension arm only visits * Follow-up required only if Screening Visit results are positive or participant is known thyroid dysfunction . ^ For females if not surgically sterile or two years post-menopause.

Visit 7 Considerations for the 16-week Endpoint.

If a participant misses 15% or more of Tofacitinib doses (3 or more pills) in the 7 days before a scheduled Visit 7 for any reason, Visit 7 will be rescheduled to ensure 7 days of compliant study drug intake before this endpoint visit. This may be outside the original +/- 3 days window for Visit 7. Treatment will not be extended beyond 18 weeks before a Visit 7 occurs. This will be recorded as a protocol deviation.

Similarly, if a participant reports a confounding adverse event within 7 days of Visit 7, Visit 7 will be rescheduled so that at least 7 days have passed since the condition was resolved. This may be outside the original +/- 3 days window for Visit 7. Treatment will not be extended beyond 18 weeks before a Visit 7 occurs. This will be recorded as a protocol deviation.

Extension Arm – Study Drug.

Some participants may not respond, or may not achieve a full response, to Tofacitinib by the end of the 16-week trial. Participants who are likely to benefit from an extension of Tofacitinib will be initially identified by the study doctor at their 8-week visit (Visit 5) using the criteria below, but may be identified as a candidate for the extension trial at subsequent visits at the study doctor's discretion if the criteria are met. Additionally,

participants must remain in good health standing and have no study doctor concerns that would preclude study continuation.

1. Alopecia areata (min. initial qualifying SALT score ≥ 25): Participant shows any decrease in their SALT score from the Baseline score, but SALT score is still ≥ 5 at the 8-week appointment.
    - In cases of alopecia totalis, observable improvement in any hair re-growth on the body would constitute a basis for Extension Arm qualification, such as eyelash or eyebrow re-growth.
    - All cases of alopecia areata present for ≥ 5 years by parent or clinician report will be invited to participate in the Extension Arm, even if no improvement decrease in SALT score is noted by 8 weeks.
2. Psoriasis (min. initial qualifying PASI score ≥ 10): Participants showing any decrease in their PASI score from Baseline, but PASI score is still ≥ 2 at 8-week appointment.
    - Participants with a diagnosis of psoriatic arthritis will be referred to a rheumatologist for on-label prescription.
3. Atopic dermatitis (min. initial EASI qualifying score ≥ 16): Participant showing any decrease in EASI score from Baseline, but EASI score still ≥ 5 at 8-week appointment.
4. Hidradenitis suppurativa (min. initial HS-PGA score ≥ 3): Participant showing a HS-PGA score ≥ 2 from Baseline with any concurrent decrease in Modified Sartorius Scale (MSS) at 8-week appointment or subsequent appointment. As HS may not respond during an 8-week course, all participants with HS may be invited to participate in the Extension Arm at the study doctor's discretion.
5. Vitiligo (min. initial qualifying VETI score ≥ 2): As vitiligo may not respond during an 8-week treatment course, all participants with vitiligo may be invited to participate in the Extension Arm at the study doctor's discretion.

Research Labs Collected	Version Date Implemented	Screening Visit 1	Baseline Visit 2	2-week Visit 3	4-week Visit 4	8-week Visit 5	12-week Visit 6	16-week Visit 7	2-week post Visit 8%	28-week Visit E28	40-week Visit E40
1) Thyroperoxidase (TPO)<br>2) Thyroglobulin (TG)<br>3) Thyroid stimulating hormone receptor (TRAb)	02.20.2020	X	X	*	N/A	*	N/A	X	*	*	*
tissue trans-glutaminase (tTG) IGA	04.22.2020	X	X	*	N/A	*	N/A	X	*	*	*
anti-deamidated gliadin peptide (DGP)	04.22.2020	X	X	*	N/A	*	N/A	X	*	*	*
Immunoglobulin A	04.22.2020	X	X	*	N/A	*	N/A	X	*	*	*
Skin tapes	02.20.2020<br>11.12.2020	N/A	X	N/A	N/A	X	N/A	X	N/A	N/A	E
Tongue swab	02.20.2020	X	X	X	N/A	X	N/A	X	X	E	E
Skin photos	02.20.2020<br>11.12.2020	X	X	$	$	X	$	X	$	$	E
ESR	11.12.2020<br>05.26.2021	X	X	X	X	X	X	X	X	E	E
CRP	05.28.2021	X	X	X	X	X	X	X	X	E	E
DNA	04.18.2022	N/A	X	N/A	N/A	N/A	N/A	X	N/A	N/A	X

Table 3. Schedule of research labs. 'X'= Test performed; N/A = Not applicable; * Follow-up required only if Screening Visit results are positive; % This visit only occurs for participants who ended Tofacitinib treatment at Visit 7; E = Extension Arm only visits; $ As needed to document skin changes

Participants will return for their 16-week visit (Visit 7) to collect the endpoints as described above. However, instead of entering a washout period after Visit 7, the participant will continue taking the IP and will return for additional visits at 28 weeks (Visit E28) and 40 weeks (Visit E40). Participants will receive an additional prescription for Tofacitinib at Visit 7 and Visit E28. Both safety labs and research labs will be collected at all Extension Arm Visits. Female participants who do not have permanent birth control will be required to take an at-home pregnancy test during the months they are not seen in clinic. Tests may be provided by the study or cost reimbursed with receipt.

The above criteria describe the minimum requirements for inclusion in the Extension Arm – Study Drug phase. Invitation to participate will continue to be subject to the study doctor's discretion and available funding for Tofacitinib.

Extension Arm – Monitoring only.

Participants may move to clinical care to receive Tofacitinib at any time, and the study team may actively facilitate this transition. If the participant begins to receive the study drug by prescription from their clinical provider, they may still remain in the Monitoring Only Extension Arm of the study. Participants will attend study visits as described above, but do not receive Tofacitinib through the clinical trial and are not subject to the inclusion criteria described above. Study participants will be asked to complete all study procedures as described, including monthly phone check-ins and medication record logs, although the Tofacitinib will no longer be managed by the Investigational Pharmacy or study team.

Washout Period.

The washout period, and collection of samples two weeks after ending the study medication (Visit 8), may be removed for all participants who continue taking Tofacitinib, either through the Extension Arm or by prescription with their personal doctor. Participants who live outside of the Denver Metro Area may opt out of returning for this study visit as well.

Additional Visits.

At any time during the study, study doctors may request an additional study visit with a participant to address or follow-up on pressing concerns about safety or reported AEs. In this case, a study coordinator will schedule an additional study visit with the participant and their study partner as soon as possible. This visit may include collection of vitals, physical exam and recording of AEs as necessary, as well as any laboratory tests recommended by the study doctor.

Teleresearch Visits.

To facilitate participation for non-local participants, study doctors may perform a teleresearch visit using Epic. These visits may be implemented at the study doctor's discretion in place of regularly scheduled visits and combined with a remote blood draw to obtain safety labs as needed. Creatine kinase is not collected for teleresearch safety labs. Research labs may be drawn remotely and shipped to the Linda Crnic Institute. Additional study drug may be dispensed at study doctor's discretion at a prior visit in anticipation of a scheduled teleresearch visit, or shipped directly to the participant from the Investigational Pharmacy.

Remote Blood Draws.

For standard safety monitoring, it may be more convenient for the participant to have laboratory tests drawn nearer to their home. The study team may arrange for a qualified phlebotomist to visit the home and subsequent clinical analysis at an accredited lab, or for the participant to go to an alternate facility for a blood draw at their discretion. Participants may always choose to have appointments at their regular study site.

COVID-19 vaccinations and boosters.

Participants who receive a COVID-19 vaccination or booster will be advised to stop taking the study drug for 7 days (14 doses) after receiving the vaccination or booster. The original study timeline will not be altered,

unless suspension of the study drug interferes with a study endpoint (see Visit 7 Considerations for the 16-week Endpoint).

Safety and Adverse Event Monitoring.

An adverse event (AE) is any untoward medical occurrence in a participant during participation in the clinical study or with use of the experimental agent being studied. An adverse finding can include a sign, symptom, abnormal assessment (laboratory test value, vital signs, electrocardiogram finding, etc.), or any combination of these. All AEs will be classified using two criteria: 1) severity, and 2) the likelihood that they are attributed to the study intervention. Additionally, regardless of its severity and attribution classifications, an AE may also be considered “serious” (SAE) and/or an “unanticipated problem” (UAP) if it meets certain criteria. These classifications and criteria are explained in depth below. The severity of AEs is based on their impact on the participant. An AE will be termed: 1) Mild: event has minimal impact on the participant, 2) Moderate: the event causes the participant some inconvenience, 3) Severe: event causes a substantial disruption to the participant’s well-being.

AE/SAEs/UAPs will also be categorized according to the likelihood that they are attributed to Tofacitinib, a classification which must be designated by a study doctor. Specifically, AEs will be categorized as: 1) Definitely related: AE is clearly related to the investigational agent, 2) Possibly related: AE may or may not be related to the investigational agent, 3) Not related: AE is clearly not related to the investigational agent.

An AE is considered unexpected if its nature, severity, or frequency is: 1) not listed in the investigator’s brochure, this study protocol, or informed consent form; and 2) not consistent with the expected natural progression of any underlying disease, disorder, or condition the participant has, including their presenting skin condition and DS.

Finally, any AE, regardless of the above classifications, can also be labeled as a serious adverse event (SAE). An SAE is any AE that results in: 1) death, 2) a life-threatening event, 3) inpatient hospitalization or prolongation of existing hospitalization, 4) a persistent or significant disability/incapacity, 5) a congenital anomaly or birth defect, or 6) an important medical event based upon appropriate medical judgment.

All AEs are recorded from the Baseline visit (Visit 2) when the IP begins through the final follow-up visit 2 weeks after the last Tofacitinib dose, or final visit of the Extension Arm. Additionally, AEs that occur at Screening (Visit 1) that are considered to be directly related to the screening procures will be reported. AEs are documented in terms of a medical diagnosis. When this is not possible, the AE is documented in terms of signs and/or symptoms observed by the study doctor or reported by the participant. AEs are recorded in REDCap and printed copies of the completed REDCap forms are kept in the participant’s source documentation file. AE incidence rates (number of events per 100 patient years) for the study as a whole will be calculated for monthly reports (see *Safety and Adverse Event Reporting* below).

In published studies evaluating Tofacitinib for treatment of rheumatoid arthritis, the incidence rate was as follows for various AEs: 9.4 for SAEs, 2.7 for serious infection, 3.9 for herpes zoster (shingles), 0.3 for opportunistic infection not including tuberculosis, 0.2 for tuberculosis, 0.9 for malignancies, and 0.1 for gastrointestinal perforations. These AE rates are anticipated by this study protocol and have already been deemed safe by the FDA via their approval of Tofacitinib for marketing.

When any AE, regardless of classification, is reported by a participant to any study staff, that personnel will immediately relay the AE information to a study doctor, who will classify the AE appropriately and decide if any follow-up actions should be taken. If needed, follow-up appointments will occur in a timely manner as dictated by the nature of the AE and the discretion of the PIs and study doctors. Similarly, all blood chemistry and laboratory monitoring values will be reviewed as soon as they are available by a study doctor to ensure all results are within normal ranges for a person with Down syndrome or for that specific participant. Abnormal findings will be reported as an AE as appropriate. Detailed information about every AE will be incorporated into a Monthly Progress and Safety Report, as well as Annual Reports, as described below.

Finally, any incident, experience, or outcome may be considered an unanticipated problem (UAP) if it meets the following criteria: 1) it is considered “unexpected” as defined above, 2) it is considered possibly or

definitely related to the investigational agent, and 3) it suggests that the study places participants at a greater risk of harm (including physical, psychological, economic, or social harm) than was previously recognized. Some AE/SAEs may also be flagged as UAPs, if all three of these criteria are met; however, UAPs can also include study-level events or outcomes encompassing more than one AE or participant.

When any SAE or UAP is discovered by a study coordinator or doctor, that personnel will immediately notify all three study PIs. The contact PI, Joaquin Espinosa, will be responsible for prompt reporting of any SAE or UAP to the appropriate agents as follows:

1) **Fatal or life-threatening SAE:** PIs notify COMIRB, NIAMS, and the DSMB within 48 hours.
2) **Possibly or Definitely related to Tofacitinib SAE:** PIs notify COMIRB, NIAMS, and the DSMB within 48 hours.
3) **All other SAEs:** PIs notify NIAMS and the DSMB within 48 hours and COMIRB within 5 days.
4) **Any UAP:** PIs notify NIAMS and the DSMB within 48 hours and COMIRB within 5 days.

<u>Safety and Adverse Event Reporting.</u>

Monthly Progress and Safety Reports will be compiled by the clinical coordination team for review by the appropriate parties. Progress and Safety Reports will include information regarding recruitment and enrollment status, comparison of targeted to actual enrollment, retention status, and demographic information of participants. These reports will also detail participant adherence to study protocol, which will be evaluated by verbal reports of pill counts at each study visit. Finally, Monthly Progress and Safety Reports will contain summarized AE information including: 1) a list of all AEs reported, including their classifications, actions taken, and outcome 2) a list of all symptoms and their frequency reported by participants, 3) all incidences of laboratory values considered out-of-range for an individual with Down syndrome, 4) a list of all SAEs reported, including their classifications, actions taken, and outcome, and 5) information about any participant deaths, including the cause of death. Monthly Progress and Safety Reports will not include subject- or group-identifiable material. Each report will only include the Study ID.

Furthermore, at the end of each study year, the PIs and Clinical Coordination Team will work together to produce an Annual Report. Annual Reports will include (1) a summary of enrollment and retention and participant demographics; (2) a summary of participant adherence to the study protocol; (3) a complete list and overall summary of all AEs; (4) an analysis addressing whether AE rates are consistent with pre-study assumptions; (5) reasons for dropouts from the study; (6) whether all participants met entry criteria; (7) whether continuation of the study is justified on the basis that additional data are needed to accomplish the stated aims of the study; and (8) conditions whereby the study might be terminated prematurely. Annual Reports will not include subject- or group-identifiable material. Each report will only include the Study ID. The Annual Report, and the DSMB report, will also be submitted to COMIRB at the Continuing Review.

Additionally, participants will be notified of UAPs that affect the level of risk of participation, and so result in changes to the protocol or consent, at the time of re-consent for active participants. Participants who have completed active trial participation will be notified in compliance with COMIRB or the NIAMS corrective action plan.

NIAMS will convene a Data Safety and Monitoring Board (DSMB) for this study. DSMB members will have no direct involvement with the study PIs or the investigational agent, Tofacitinib. The DSMB will act in an advisory capacity to NIAMS who monitors participant safety and progress of the study, and review procedures for maintaining data confidentiality, quality, management, and analyses.

<u>Participant Discontinuation and Trial Stopping.</u>

Study doctors, at their discretion, may discontinue Tofacitinib, temporarily or permanently, and/or may remove participants from the study.

Specific trial stopping rules for individual participants are as follows:

1) Participant decides to discontinue participation for any reason.

2) Participant experiences adverse or allergic reaction to Tofacitinib, as determined by the study doctor.
3) Participant experiences an AE that precludes trial completion, as determined by the study doctor.
4) Clinically significant abnormal lab finding, as determined by the study doctor. Abnormal lab findings will be repeated within one week to confirm.
5) Participant becomes pregnant.
6) Any medical or personal event(s) or participant non-adherence resulting in missed Tofacitinib doses totaling 15% (34 doses) or more of total study drug for the 16-week treatment period (224 doses). At the Endpoint Visit (Visit 7), this calculation will be re-applied with the true number of doses the participant had the opportunity to take within the allowable +/- 3 day study visit window (e.g., 221 or 222 doses for a participant whose Endpoint visit is scheduled one day earlier than 16 weeks) to confirm the adherence rate for the actual treatment period. For participants in the Extension Arm, participants missing more than 15% of total doses at E28 will be terminated from the study.
7) Two missed study visits without study doctor approval.
8) If at any point during the study a participant fails to return communication with a study coordinator after 3 consecutive attempts made using at least 2 forms of contact provided by the participant and/or study partner, then Dr. Espinosa will make at least one attempt to contact the participant using their preferred method of contact. After a total of five unreturned communications, the participant will be considered lost to follow-up and no further contact attempts will be made.
9) A study doctor may discontinue a participant from Tofacitinib for any reason, including noncompliance, at the study doctor's discretion. It should be noted that discontinuation of Tofacitinib, for whatever reason, does not necessarily constitute withdrawal from study. A study doctor, in consultation with all PIs, may advise a participant and their study partner to discontinue Tofacitinib temporarily while remaining in the study.
10) Alternatively, a study doctor may decrease the dosage to 5 mg once daily. These determinations will be considered by the PIs on a case-by-case basis.
11) Participant experiences an SAE definitely related to Tofacitinib.

Participants who are removed from study for reasons other than an adverse drug reaction may, if they choose, return and start the entire study over again (beginning with the Pre-screening phone interview or a Screening visit, as appropriate), at the discretion of study staff and after at least 21 days have passed since taking their last dose of Tofacitinib.

This entire clinical trial, including the Extension Arm, will be stopped prior to its completion if:

1) the intervention is associated with adverse effects that call into question the safety of the intervention;
2) difficulty in study recruitment or retention will significantly impact the ability to evaluate the study endpoints;
3) any new information becomes available during the trial that necessitates stopping the trial; or
4) other situations occur that might warrant stopping the trial; or
5) more than two SAEs that are definitely related to Tofacitinib are reported within a 14.5 patient year trial period; or
6) the rate of SAEs definitely related to Tofacitinib per 100 patient years exceeds 13.83 at any time after least 14.5 patient years have occurred (see *Research Methods for Specific Aim 1* for calculations).

The PIs will perform a qualitative interim analysis after recruiting and completing all clinical and data generation activities on at least the first 10 participants and will consult with NIAMS, the DSMB, and experienced clinical trial statisticians to assess the impact of significant data loss due to problems in recruitment, retention, or data collection. Up to 15 participants may be included in the interim analysis to account for recruitment surges and possibility of attrition.

<u>Incidental Findings.</u>

Incidental findings will be handled in accordance with COMIRB policy and the appropriate language regarding the possibility of incidental findings will be included in the informed consent form. The potential return of any clinically confirmed incidental findings will be considered on a case-by-case basis in consultation with study

doctors, COMIRB, the DSMB, and an ethics officer from the University. If the findings are returned to the participant, the study doctors would provide appropriate resource recommendations to the participant for understanding and dealing with the findings. The costs for any care that will be needed to diagnose or treat an incidental finding would not be paid for by this research study and would be the responsibility of the participant.

Study doctors may request additional blood tests or other assessments or consultations before determining eligibility based on screening results. These additional eligibility determination tests, prior to enrollment at Visit 2, are not considered adverse events or Incidental Findings.

Return of Results.

Participants can request a copy of their cognitive test results and/or karyotype and/or chest x-ray at the completion of the R61 phase of the study. Copies of their audiogram results may be provided at the time of assessments. Chest radiographs and audiogram results will become part of the participant's clinical history in the electronic medical record (EMR). Safety and monitoring labs performed by a CLIA-certified laboratory (e.g. University Hospital) will become part of the participant's EMR and released to the participant, and may be released to a provider of participant's choosing, at the participant's request, to facilitate clinical care. De-identified analyses and aggregated study results may be communicated to participants via newsletters or other media.

Research Methods for Specific Aim 1. To define the safety profile of JAK inhibition in Down syndrome.

Although several JAK inhibitors are FDA-approved and widely used in the typical population, it is possible that JAK inhibition may have a different safety profile in people with DS. Thus, a primary endpoint of our trial is to define safety in those with T21. Among JAK inhibitors, Tofacitinib has by far the most available safety data, and the most extensive testing on immune skin conditions (38-44). Furthermore, we are encouraged to employ Tofacitinib by the fact that one of our research participants with DS has been taking this drug for nearly four years without adverse effects. Another reason to employ Tofacitinib is that it is a JAK1/3 inhibitor, with minor activity toward JAK2, which improves its safety profile (108), yet it would also inhibit accompanying inflammatory pathways elevated in DS that required JAK1/3 (e.g. IL6 signaling) (108). Tofacitinib can be purchased by the research team, thus providing autonomy in the design of the trial. Finally, our dermatology study doctors have extensive experience with Tofacitinib.

As described above, our study doctors will monitor AEs and assign a likelihood that they are attributable to Tofacitinib treatment. Published safety data for Tofacitinib for treatment of RA in typical people indicates 9.4 serious adverse event (SAEs)/100 patient years (all SAEs, treatment-related or not) (109). Given that our trial aims to enroll 47 participants for 16 weeks of treatment (though we anticipate up to seven participants would be lost to attrition at various times during the treatment course) for a maximum total of 14.5 patient years of treatment, a comparable number of SAEs is 1.36. Accordingly, and with the knowledge that our population may be more prone to AEs in general without any treatment due to other health conditions, we define the success of our primary endpoint associated with this aim as no more than two SAEs *definitely related* to treatment during the 16-week trial.

For the purposes of ongoing safety monitoring during the optional Extension Arm and after the initially planned 14.5 patient years of treatment time, we will transform this endpoint of "no more than two SAEs definitely related to treatment during the 16-week trial" to an allowable *rate* and apply the same criteria for overall stopping rules. Accordingly, 2 allowable SAEs definitely related to Tofacitinib over the course of 14.5 patient years translates to a rate of 13.83 SAEs/100 patient years (see stopping rules #5 and #6).

Research Methods for Specific Aim 2. To determine the impact of JAK inhibition on the immune dysregulation caused by trisomy 21.

Our results demonstrate that individuals with T21 show obvious signs of immune dysregulation and hyperactivation of IFN signaling at the levels of mRNA signatures, cytokine profiles, and plasma metabolites. In this Specific Aim, we will define whether these inflammatory markers can be driven by Tofacitinib toward values observed in typical people. Importantly, we have already defined the directionality and average

magnitude of the change between people with and without T21 (*Figures 3-5*). For example, we determined that IFN scores are significantly higher in people with DS (*Figure 3C*) and expect that Tofacitinib will reduce these values. Whereas some of the features dysregulated by T21 may be normalized by Tofacitinib treatment, others may not, and this will provide important mechanistic insights into the molecular underpinnings of immune dysregulation in DS. Therefore, in this Aim we will investigate the impact of Tofacitinib on IFN transcriptional scores derived from white blood cells (WBCs) (primary endpoint), levels of circulating inflammatory cytokines (secondary endpoint), levels of neurotoxic metabolites in the IFN-inducible kynurenine pathway, and key autoantibodies associated with AITD and celiac disease (tertiary endpoints) at baseline and 16 weeks of treatment with Tofacitinib.

IFN transcriptional scores. We will monitor the impact of Tofacitinib on WBC IFN transcriptional scores for each participant at baseline and at 16 weeks of treatment. Additional time points will be collected and processed in a fashion compatible with future analysis, and skin tape samples will be banked for future cytokine analysis. IFN scores have been developed for IFN-driven conditions such as SLE and Type I Interferonopathies (69-71), and consist of a composite Z-score that integrates mRNA expression values for multiple ISGs into a single numerical value that can be used to track correlations with disease severity (71), and which have also proven effective to monitor the impact of JAK inhibition in clinical trials (69). Using published methods (69-71) we observed significantly higher Type I IFN scores in both T cells (*Figure 3C*) and total WBCs (110). Thus, we clearly have the expertise to monitor changes in IFN signaling with this approach. Much of this protocol has been described in our previous publication (27). RNAs will be extracted using the PAXgene Blood RNA Kit and quality asses using the Agilent 2100 Bioanalyzer System. Next-generation sequencing libraries will be prepared with the Illumina TruSeq Stranded mRNA Library Prep Kit and sequenced with an Illumina NovaSeq 6000 instrument. Analysis of library complexity and high per-base sequence quality across all reads will be performed using FastQC software. Other bioinformatic steps include trimming of low-quality bases, short reads, and adaptor sequences, with the fastqc-mcf tool; removal of mycoplasma, mitochondria, and rRNA contaminant sequences with FASTQ Screen; read alignment to GRCh37/hg19 using TopHat2; filtering of high-quality mapped reads with SAMtools; and final quality performed using RSeQC. Gene level counts will be then obtained using HTSeq (111). IFN scores will be calculated by adapting methods described in the literature (69-71), using RPKM values instead of values derived from Nanostring or q-RT-PCR data. We define success of this endpoint as statistically significant reduction in average IFN scores between baseline and 16 weeks as defined by a two-sided, paired Student's t-test.

Levels of circulating inflammatory cytokines. We will monitor the impact of Tofacitinib on cytokines using multiplex immunoassays with Meso Scale Discovery (MSD) technology, which are highly efficient sandwich immunoassays used to measure the levels of multiple protein targets within a single, small-volume reaction. MSD utilizes electrochemiluminescence to detect multiple binding events simultaneously with patterned arrays. We will use a custom V-PLEX platform following standard manufacturers' protocols as in our recent publication (28). This panel consist of cytokines that are significantly elevated in people with DS, with obvious ties to IFN signaling, autoimmunity in general, and immune skin conditions in particular (*Figure 4* and data not shown). We will measure multiple cytokines, chemokines, and immune factors dysregulated in DS, including but not restricted to: IP10, TNFα, CRP, IL6, IL10, MCP1, IL2, IL22, VEGF, IL17A, IL8, MIP1α, IL29 (IFNλ), IFNγ, and IL1β. Four of these cytokines (IP10, TNFα, CRP, IL6) will be used to create a composite Z-score (*Figure 4B*) to be used in endpoint evaluation, with the other cytokines being measured for research purposes. To minimize batch effects, we will use the V-PLEX immunoassays, which are analytically validated with guaranteed performance specifications that offer consistent results over long-term studies. Each assay employs a maximum volume of 25 µL and each plasma sample is analyzed in duplicate. Plates will be read on the Meso QuickPlex SQ 120 system and data analyzed using the MSD workbench software. Absolute quantifications are determined with MSD-provided standards. We define success of this endpoint as statistically significant reduction in the cytokine score at 16 weeks of treatment as defined by two-sided, paired Student's t-test.

Levels of neurotoxic metabolites in the IFN-inducible kynurenine pathway. Plasma metabolomics will be performed at the Metabolomics Core at CU-Anschutz using established protocols and instruments as

previously reported (112). Briefly, metabolites will be extracted from plasma in acetonitrile:methanol:H_2O at a 5:3:2 ratio (v/v/v). Extraction efficiency and instrument performance will be monitored by the incorporation of internal standards such as 5-fluorouracil and $_{13}C_6$-lysine into the extraction solution, as described (113). In addition, >80 stable isotope internal standards will be added to samples for absolute quantification, including up to 12 metabolites in the KP. Metabolites will be resolved via reversed-phase (RP) and hydrophilic-interaction liquid chromatography (HILIC) using a Vanquish UHPLC (Thermo) coupled to a high-resolution quadrupole orbitrap mass spectrometer, including three QExactives, a QExactive HF, and an Orbitrap Fusion Lumos scanning in full MS mode in the 60 to 900 m/z and 100 to 1,500 m/z range, operated separately in negative and positive ion mode. Targeted data analysis will be performed using MAVEN for manual and XCMS and Compound Discoverer for automatic metabolite identification. These tools are used for peak picking and metabolite assignment using the KEGG, HMDB, and NIST pathway databases. Metabolite identifications will be further confirmed by determination of the chemical formula using isotopic patterns and high-resolution accurate intact mass and fragmentation spectra of targeted features. We will evaluate three different KP dysregulation values: absolute levels of KYN and QA, and the KYN/TRP ratio. All three values are significantly elevated in people with DS relative to typical individuals (*Figure 5* and (110)). We define success of this endpoint as statistically significant reduction in one or more of three kynurenine dysregulation values at 16 weeks of treatment as defined by a two-sided, paired Student's t-test with FDR correction.

Levels of key autoantibodies associated with AITD and celiac disease. Previous studies defined that ~ 60% of adults with DS carry anti-thyroid antibodies (9-11), and >30% carry autoantibodies linked to celiac disease etiology (18, 52, 114, 115). Thus, as part of our mechanistic investigations, we will measure levels of three key autoantibodies involved in AITD [thyroglobulin (TG), thyroid peroxidase (TPO) and thyroid-stimulating hormone receptor (TSHr)], and two autoantibodies relevant to celiac disease [anti-tissue transglutaminase antibodies (tTG) and deamidated gliadin peptides (DGP) antibodies]. All tests will be performed at the University of Colorado Health Hospital Clinical Laboratory. We define success of this endpoint as statistically significant reduction in levels of one or more autoantibodies at 16 weeks as defined by a two-sided, paired Student's t-test.

<u>Research Methods for Specific Aim 3. To define the impact of JAK inhibition on immune skin conditions in people with Down syndrome.</u>

Given the high prevalence of immune conditions in the DS population and the wealth of data suggesting this could be explained by their obvious hyperactivation of IFN signaling, we propose here to evaluate the impact of 16-week treatment with Tofacitinib on clinical improvement of immune skin conditions. These efforts will be led by a multidisciplinary team of dermatologists with extensive expertise in clinical trials for JAK inhibitors in typical people (Drs. Norris, Dunnick, and Wallace). Improvement in skin pathology will be the main secondary endpoint of this trial, defined as a statistically significant percentage reduction in average disease scores after 16 weeks of treatment.

From a dermatology standpoint, this trial is considered a 'bucket trial', so designated because we will include individuals with DS and diverse active immune skin conditions. We aim to enroll at least 10 participants during the initial phase, before performing a qualitative interim analysis and continuing with 30 additional participants. Up to 15 participants may be included in the interim analysis. Based on our ongoing cohort study and epidemiological data of skin diseases in people with DS (24, 25, 116-118), we expect to enroll five predominant conditions: AD, AA, HS, psoriasis, and vitiligo (*Figure 1B*), but will accept other immune skin conditions according to our dermatologists' expertise. Being an open-label, proof-of-concept study, all participants will receive Tofacitinib at 5mg BID for 16 weeks. We will evaluate changes from baseline to 16 weeks in: 1) Investigator's Global Assessments (IGAs), 2) Dermatology Life Quality Index (DLQI) (119), and 3) Disease-specific metric scores. The IGA and DLQI will serve as 'composite endpoints' to capture overall clinical improvement in a disease-agnostic fashion. Additionally, each participant will be monitored using the relevant accepted disease-specific metric scoring systems, for example: Eczema Area and Severity Index (EASI) for AD, Severity of Alopecia Tool (SALT) for AA, Modified Sartorius Score (MSS) for HS, Psoriasis Area and Severity Index (PASI) for psoriasis, and Vitiligo Extent Tensity Index (VETI) for vitiligo (120-124).

These measures are all well-validated and widely used in clinical trials related to skin disease, and our team of dermatologists has extensive experience with all these metrics. We define success of this endpoint as statistically significant *percentage* reduction in a) IGA values, b) DLQI values, and c) disease-specific scores between baseline and 16 weeks as defined by a two-sided, paired Student's t-test.

<u>Research Methods for Specific Aim 4. To characterize the impact of JAK inhibition on cognition and quality of life in Down syndrome.</u>

Valid assessment of the impact of JAK inhibition on cognitive and behavioral outcomes in DS will require phenotype sensitive measures, which has been a methodological challenge in previous clinical trials in DS. To address this issue, the NIH-assembled Outcomes Measures Working Group for Clinical Trials in DS recently published guidelines for behavioral and cognition measures appropriate for use in clinical trials (125). Dr. Fidler was a contributor to this Working Group and the published guidelines for more scientifically-sound measurement selection. This guidance considers critical psychometric parameters, such as the need for test-retest reliability, sensitivity to change, and construct validity for individuals with intellectual disability, and DS in particular. Based largely upon these guidelines, we selected the following measures which assess specific cognitive processes that are key components of the neurocognitive phenotype in DS, and which we hypothesize could be impacted by JAK inhibition:

a) Leiter 3
   i) Attention Sustained
   ii) Nonverbal Stroop Tasks
   iii) Forward/Reverse Memory
   iv) Sequential Order
b) CANTAB
   i) Spatial Span
   ii) Paired Associate Learning
   iii) Reaction Time
c) NEPSY II Visuomotor
d) PPVT 5 Form A
e) KBIT II
f) SOBC SRT
g) SALT Story Elicitation task

Subdomains from the Leiter-3 International Performance Scale will be used to assess executive function including inhibitory control (Sustained Attention), working memory (Forward and Backward Memory), and cognitive flexibility (Nonverbal Stroop Tasks), as well as spatial processing (Sequential Order). The Leiter 3 is a lifespan nonverbal measure of cognition, attention, and neuropsychological function and was normed on individuals ages 3 to 75 years. This measure was endorsed by the NIH Working Group (125), and it is considered to be a phenotype-sensitive measure for individuals with DS because it involves minimal receptive language, and no expressive language, demands. Subdomains from the CANTAB (126) will be used to assess episodic memory (Paired Associate Learning), spatial processing (Spatial Span), and processing speed (Reaction Time Interval). An additional Simple Reaction Time test will be administered from the science of behavioral change website. The SALT story elicitation task has been added to capture expressive language. The NEPSY II Visuomotor will assess motor control. In addition, the KBIT II and PPVT 5 are included as control measures of overall development status and vocabulary. This battery takes into consideration length of time required for administration, its suitability for individuals with varying degrees of cognitive ability, and previous experience of our team in terms of participant acceptability. This short test battery will be administered and video recorded at baseline blood draw (+/- 3 days), and again at 16 weeks of treatment and at E40 for those in the Extension Arm. We define success of this endpoint as statistically significant improvement in any one of the cognitive measures described above between baseline and 16 weeks as defined by a Wilcoxon signed-rank test with FDR correction or a two-sided, paired Student's t-test with FDR correction, as appropriate.

While not all affected cognitive skills may be increased in a measurable sense by JAK inhibition, the individual's or caregiver's perception of overall quality of life may change independent of changes in the cognitive measures proposed, either due to changes in cognitive processes not assessed as part of our trial or improvement in skin conditions as a result of treatment with a JAK inhibitor. Therefore, we will assess quality of life at baseline and again at the conclusion of treatment (16 weeks), and 40 weeks for those in the Extension Arm, using the following PROMIS metrics available through the NIH Toolbox, which are appropriate for individuals with special needs (127, 128):

a) Emotional Distress-Anxiety Short Form 4a
b) Emotional Distress-Depression Short Form 4a
c) Cognitive Abilities Short Form 4a
d) Positive Affect Short Form 4a

We define success of this endpoint as statistically significant improvement in any NIH PROMIS tool between baseline and 16 weeks as defined by a two-sided, paired Student's t-test.

D. Specific study activities implemented during COVID-19.

The following additional measures will be implemented by the study team as necessary to comply with current local guidelines during the COVID-19 pandemic. These measures are subject to change in response to State, local, and University regulations and guidance as the pandemic progresses. If any measure conflicts with direction given in this protocol or any other study document, the COVID-19-specific measure listed here will prevail.

Precautionary and Informative Measures.

1) The informed consent form has been updated to explain potential additional risks and benefits of study participation during COVID-19.
2) Study coordinators will ask participants and their study partners a series of COVID-19 screening questions during the Pre-screening phone interview to ensure they have no symptoms of and have not been in recent contact with someone with COVID-19. Coordinators should refer to the University's most recent Clinical Research Reconstitution Plan for appropriate screening questions, which can be found on the University's Weekly Update on Research Reconstitution webpage (https://www.cuanschutz.edu/coronavirus/research-guidance/updates). Anyone who positively answers any COVID-19 screening questions will have their study visit rescheduled.
3) Participants who are experiencing any COVID-19 or COVID-19 like symptoms should notify study staff and proceed in taking a COVID test. If symptoms persist and/or the patient receives a positive COVID-19 diagnosis their study visit will be rescheduled.
4) Study coordinators will inform participants and their study partners before each study visit that only they will be allowed on campus – no other friends or family may attend a study visit with them – and that they will be escorted by a study coordinator at all times while on campus.
5) Study coordinators will provide a digital, picture-based, "social story" to explain and normalize the extra precautions that are taken at the study visits (face masks, sanitizer, etc.) for study partners to review with participants at home in the days before a study visit.
6) Study staff will follow all guidance for returning to campus, including taking appropriate training, and adhering to appropriate social distancing and cleaning and sanitizing guidelines on the University's Return to Campus webpage (https://www.cuanschutz.edu/coronavirus/return-to-campus) and detailed in the approved Space Management plans for their work areas.
7) At the Baseline visit, participants will be given a "What to do if I get COVID-19" card with information for them and their physician regarding the study and potential impacts of Tofacitinib on COVID-19.
8) Only pre-packaged snacks or juice will be provided to participants during a study visit in a properly sanitized area.
9) When preparing banked blood samples for Research Methods for Specific Aim 2, a PCR and/or antibody test for SARS-CoV-2 may also be performed, allowing the team to analyze prior or current SARS-CoV-2 infection as a potential confounding variable or co-variate during immune dysregulation and biomarker analyses.

10) Participants will be advised to receive all vaccinations, including for COVID-19, before beginning tofacitinib. However, if they are already taking tofacitinib, they may be advised to withhold tofacitinib for a period of time before and/or after receiving a COVID-19 booster, in accordance with American College of Rheumatology (ACR) guidance. Current ACR guidelines recommend holding tofacitinib for 7 days (14 doses) after vaccination. When appropriate amid changing guidelines, study doctors will encourage participants and their study partners to pursue boosters in accordance with best practice guidelines, even if it results in time off tofacitinib during the study period.

<u>COVID-19 Symptoms, Diagnosis, or Exposure.</u>

If at any point in the study period, a participant and/or their study partner is not allowed on campus due to COVID-19 exposure, symptoms, or diagnoses, the workflows detailed in **Figure 7** below will apply, depending on when in the study the COVID-19 event is noted by a study coordinator.

Participants who are diagnosed with COVID-19 will be reminded of their “What to do if I get COVID-19” card and instructed to bring this information to their treating physician. The participant and their physician will decide whether the participant should continue taking Tofacitinib and remain enrolled in the study. In this scenario, study PIs will be available for consultation with the participant’s physician. The study team will not provide any COVID-19 care but will continue to provide Tofacitinib as long as the participant remains enrolled. Study coordinators may make additional medical record requests to capture relevant information about the illness if the participant remains enrolled. Any COVID-19 diagnosis will be recorded an as AE and treated as

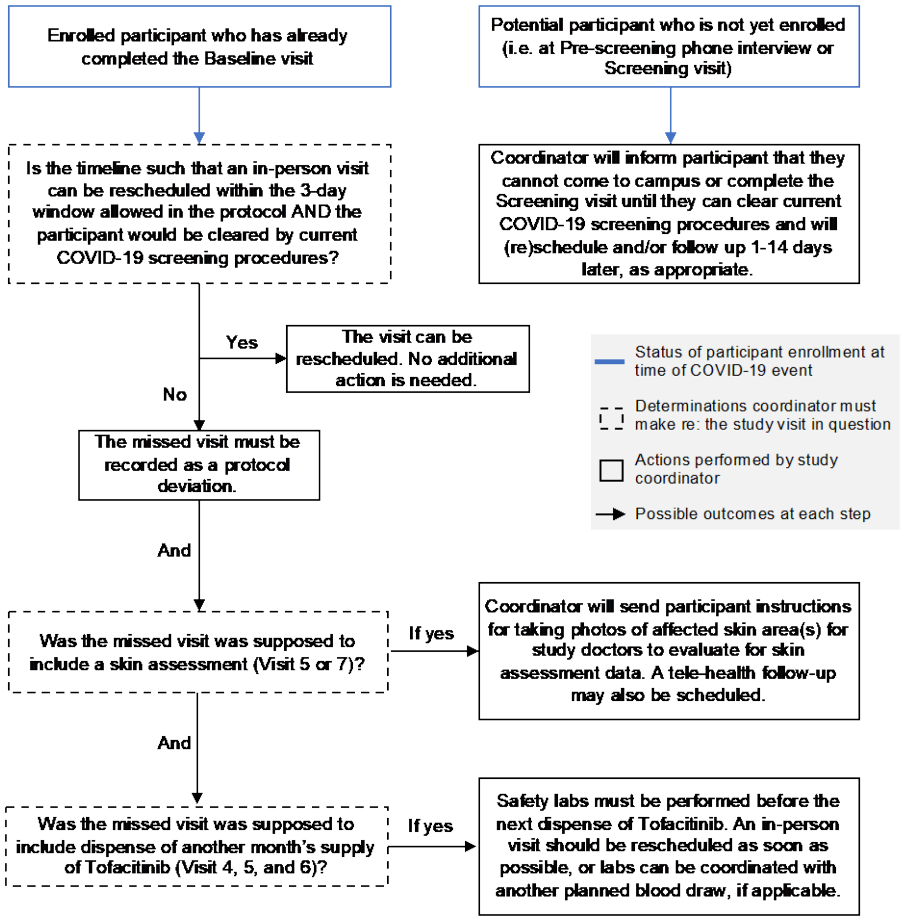

Figure 7. Flowchart detailing how study coordinators will handle any participant or study partner with COVID-19 symptoms, diagnosis or exposure.

any other infection would be, with study staff following all rules detailed in the *Participant Discontinuation and Trial Stopping* section of the protocol.

V. Description, Risks and Justification of Procedures, and Data Collection Tools

Description of Procedures.

The bulk of all clinical activities and study visits will take place at the CU Medicine Colorado Translational Research Center (CTRC). The CTRC provides private, quiet exam rooms to CU-Anschutz researchers for free. Audiograms and chest x-rays will be performed at the UCH Hospital on the CU-Anschutz campus. Cognitive assessments will be performed in a private room at the Linda Crnic Institute. Clinical labs and autoantibody assessments will be performed at the UCH Clinical Laboratory. All researcher labs, offices, and facilities involved in the clinical trial are located at CU-Anschutz and are within a five-minute walk of each other. Blood draws will be performed by qualified phlebotomists who have extensive experience with participants with DS. Participants and their representatives are accompanied to the CTRC by a clinical coordinator, who will then communicate with a member of the laboratory team for the timely transfer of the tubes containing the blood.

In addition to the study visits and activities and AE monitoring described in detail above, existing medical records will also be accessed during the trial. All data will be given an alphanumeric, randomly generated Study IDs and will be stored in HIPAA-compliant password and access restricted programs, such as REDCap. The linker key for identification of participant names corresponding to Study IDs will be access restricted to select members of the clinical team who need this information for job related duties. As such, the resulting coded information available to investigators is considered de-identified in accordance with HS.gov guidance (https://www.hhs.gov/hipaa/for-professionals/privacy/special-topics/de-identification/index.html#specificstudies). This study will use University mandated web-based study scheduling module called Oncore, that uses participant level identifiable information to integrate scheduling, financial, and study visit tracking information.

From coded blood samples, our laboratory team will separate plasma, white blood cells (WBCs), and red blood cells (RBCs), and will generate various molecular and cellular datasets, including transcriptome data from WBCs (RNA-seq), cytokine data (MSD assays), metabolomics data, and autoantibody data. All coded clinical and molecular data will be stored in HIPAA-compliant a central data repository hosted by the CU-Anschutz School of Medicine Information Technology Office.

Risks, Protections from Risk, and Justifications.

The potential risks associated with participation in the study center largely around the usage of Tofacitinib. Increased risks associated with taking Tofacitinib include: development of serious infections and sometimes fatal infections, especially in participants taking concomitant immunosuppressants; development of solid tumor cancer; laboratory abnormalities such as lymphocytosis, neutropenia, anemia, elevated liver enzymes, elevated lipids, elevated serum creatinine, and elevated blood creatine phosphokinase; nasopharyngitis; diarrhea; headache; rash; nausea; and hypertension. Additional risks may include gastrointestinal perforation. These risks will be addressed in the informed consent form.

To mitigate these risks, the design of the inclusion and exclusion criteria, as well as the study visit timeline and safety monitoring labs, have been optimized to meticulously protect participants. Individuals with any active infection and those taking concomitant immunosuppressants will be excluded from the study due to the increased risk of developing severe infections. Individuals with any history of malignant solid tumor cancers will be excluded from the study due to the increased risk of developing a malignancy. Safety monitoring will be performed at each visit to ensure participants do not develop infections or unsafe levels of cell counts, liver enzymes, lipids, creatinine, and creatine phosphokinase. Participants will see a study doctor at each of the eight visits to monitor overall health and ensure any other side effects, such as headache, nausea, diarrhea, or high blood pressure are appropriately managed and reported.

Because development of infection is a risk associated with Tofacitinb, it should be noted that individuals with DS have higher incidence and severity of some specific infections, especially respiratory tract infections due to respiratory syncytial virus and *S. pneumonia* (129-132). A variety of factors contributing to this increased risk have been studied, including immune deficiencies and anatomical abnormalities in the airway and ear, though none have yet translated to clinical improvements in morbidity and mortality. An interesting hypothesis with regard to the increased *bacterial* infection with *S. pneumonia* being tested now by our group is that constitutive activation of IFN signaling in the DS lung drives immune suppression and predisposes individuals with DS to infection, much like the epidemiological observations in typical individuals that susceptibility and severity of *S. pneumoniae* infection increases secondary to a course of viral infection, such as influenza (133-135). If this hypothesis were correct, we could reasonably expect an *improvement* in bacterial infection risk with Tofacitinib treatment, though this may or may not be observed during the 16-week treatment in this clinical trial. Nevertheless, though it may seem initially counterintuitive, precedent does exist for treating participants already at-risk for infection with immunosuppressive therapies, as is the case for systemic lupus erythematosus (SLE), another IFN-driven condition (136). Multiple therapeutic strategies have been used to inhibit IFN signaling in SLE, such as anti-IFNR antibodies (30), an IFN 'vaccine' (34), and JAK inhibitors, including Tofacitinib (35-37, 137). Given these factors, and the preventative design of the trial described above, we believe the potential for increased risk of infection in the population with DS when treated with Tofacitinib to be within reason and well-mitigated.

Outside of the risks associated with Tofacitinib, additional risks associated with participation in the clinical trial are minimal and include the risk of anxiety, fatigue, boredom or frustration during the completion of the questionnaires and testing sessions, and the potential loss of confidentiality as a result of a security breach to access identifying clinical data. In addition, participants in the clinical trial will be asked to give blood, with the implied potential risks of venipuncture, including potential bruising or minimal scarring of the skin at the site of the puncture. The potential minimal risk of the loss of privacy will be addressed in the consent form.

To protect from these minimal risks, the study will be explained in a clear and detailed manner by a clinical coordinator prior to enrollment. Participants will be given opportunities to ask questions and will be informed that they can drop out of the study at any time for any reason or for no reason. A signed copy of the consent form will be given to each participant. During the cognitive testing sessions, participants will be given the opportunity for frequent breaks as needed. If necessary, longer study visits which include cognitive testing will be broken down into two shorter visits or rescheduled in order to accommodate the participants' comfort. If participants express fatigue or discomfort, they will be given the option to stop participation or to have adjustments made as needed.

The potential benefits of participating in this clinical trial to an individual with DS are great and diverse. Due to JAK inhibition with Tofacitinib, participants may experience improvement in their presenting immune skin condition, including but not limited to AD, AA, HS, vitiligo, and psoriasis. Participants may also experience improvement in a range of other immune conditions common in individuals with DS, such as hypothyroidism, celiac disease, and type I diabetes, as well as improvement in cognition. In addition to these, participants may feel a sense of pride having been able to contribute to meaningful research that one day may help themselves or others with DS. All efforts have and will be made by the study team to minimize potential risks to participants while maximizing potential benefits. In our opinion, the far-reaching potential health benefits of participation in the clinical trial outweigh the potential risks, which are closely monitored and carefully managed by the study team.

VI. Potential Scientific Problems:

Specific Aim 1. Despite the FDA-approval of Tofacitinib for diverse immune conditions and its widespread testing in the typical population, it remains possible that it may cause more SAEs in people with DS, in which case we would stop this trial. A *qualitative* interim analysis, including all AE data, after completing all trial activities on at least 10 participants, and up to 15 enrolled participants to account for attrition, will help to inform early indication of safety and whether to proceed with the rest of the trial. If Tofacitinib proves to be more unsafe in DS, in the future (outside of the scope of this protocol), an alternative could be one of the newly developed JAK1-specific inhibitors that are currently being tested in >10 clinical trials for immune skin

conditions. Another potential problem could be to recruit 40 participants as a single site, the likelihood of which will be defined at the end the qualitative interim analysis. If a multi-site trial is deemed necessary to complete the trial, we would work with COMIRB to alter our protocol accordingly and establish partner sites, pending funding. If this were the case, our team has established collaborative relationships with other DS clinics in the United States, including the Adult Down Syndrome Clinic at Advocate Lutheran General Hospital, Park Ridge, IL (via Dr. Brian Chicoine); the Down Syndrome Clinic and Research Center at Kennedy Krieger Institute, Baltimore, MD (via Dr. George Capone); and the Developmental Disabilities Practice-Based Research Network in Cleveland, Ohio (via Dr. Carl Tyler), which could potentially serve as additional sites.

Specific Aim 2. As demonstrated by our preliminary data and publications, we have strong expertise in the research activities proposed, and do not foresee major issues with data generation. In terms of data analysis, as an alternative to the *univariate* approach described below in *Data Analysis Plan*, we could embark on *multivariate* machine learning approaches, such as random forests and support vector machines (e.g. (138, 139)), to identify *combinations* of ISGs, cytokines, or metabolites, that change significantly upon Tofacitinib treatment. These efforts will be for research purposes only, and not part of our endpoints.

Specific Aim 3. It is possible that some skin conditions will be more sensitive to JAK inhibition than others. For example, in our experience, AD, the most common of the evaluated conditions among those with T21, is more likely to respond rapidly to JAK inhibition than vitiligo. If this proves to be the case during the initial 10-participant phase, we may revise our inclusion criteria for the remainder of the trial to maximize recruitment of individuals that will experience a clinical benefit within 16 weeks of treatment. It is also possible that we will have responders and non-responders even within the same immune skin condition. Although this would dilute our chances of reaching our endpoints, it could be nonetheless very informative to identify differences in IFN scores, cytokines, metabolites or other clinical variables among the two groups, which would inform the design of follow up trials.

Specific Aim 4. It is possible that changes in cognition may not be discerned during the relatively short time course of treatment. Also, it is possible that not all participants will be able to complete all tasks. However, we have taken steps to minimize these risks associated with a short treatment window by selecting measures that are scalable and highly sensitive to change. We have addressed the attrition concern by selecting measures that are developmentally engaging, brief, and easy to administer, thus reducing threats to validity based on participant motivation issues. Furthermore, to address confounding issues observed in previous DS assessment work, each task specifically minimizes motor, expressive language, and receptive language demands, all areas of pronounced challenge in DS. The proposed assessments, therefore, strike a balance between the most advanced approaches to characterizing cognition while maintaining a high degree of feasibility of administration and minimizing phenotype specific threats to validity and reliability. Nevertheless, during the initial 10-participant phase we will explore other instruments driven by new findings related to DS outcome measure validation.

VII. Data Analysis Plan:

Specific Aim 1. To define the safety profile of JAK inhibition in people with Down syndrome.

Primary Endpoint: Evaluate the safety of Tofacitinib in people with Down syndrome.

Endpoint Definition: no more than two serious adverse events (SAE) *definitely* related to treatment during the 16-week study period (see *Data and Safety Monitoring Plan*).

Method: This number is based on published safety data for Tofacitinib for the treatment of rheumatoid arthritis in the general population, which indicates a rate of 9.4 SAEs per 100 patient years. Given that our trial aims to enroll 47 participants for 16 weeks of treatment (though we anticipate up to seven participants would be lost to attrition at various times during the treatment course) for a maximum total of 14.5 patient years of treatment, a comparable number of SAEs is 1.36.

Specific Aim 2. To determine the impact of JAK inhibition on the immune dysregulation caused by trisomy 21.

The overall goal of our data analysis plan in this Aim is to define which immune processes dysregulated by T21 can be normalized by Tofacitinib treatment, i.e. returned to values observed in typical people. Importantly, data shown in this proposal demonstrate that we have already defined the directionality and average magnitude of the change between people with and without T21. For example, we determined that IFN scores are significantly higher in people with DS (*Figure 3C*) and expect that Tofacitinib will reduce these values. Whereas some of the features dysregulated by T21 may be normalized by Tofacitinib treatment, others may not, and this will provide an important mechanistic insight to understand the molecular underpinnings of immune dysregulation in DS. Accordingly, the endpoints associated with this Specific Aim focus on effects of Tofacitinib (16 weeks of treatment) on four classes of molecular markers of immune dysregulation:

Primary Endpoint: Change in IFN scores in the transcriptome of white blood cells.

Endpoint Definition: Statistically significant reduction in average IFN scores between baseline and 16 weeks as defined by a two-sided, paired Student's t-test.

Method: To determine the impact of Tofacitinib on IFN scores, a two-sided, paired Student's t-test will be used to compare baseline and 16 weeks of treatment for participants with good compliance with the study drug (2 or fewer missed pills) and without a confounding reported adverse event in the 7 days prior to the Visit 7 endpoint. With 40 participants, we have 80% power to detect an effect size of 0.45 with a p-value <0.05, as calculated by G*Power software.

Exploratory Research Goal: A more exploratory research goal, not considered part of the primary endpoint, would be to define if there are differences in IFN scores between clinical 'responders' and 'non-responders' as defined by changes in IGA, DLQI, or skin-specific scores as defined in Specific Aim 3 below. It is difficult at this point to estimate how many responders versus non-responders there will be in the study, and hopefully, all participants will see clinical improvement, in which case this exercise will not be necessary. Assuming that half of the participants will see clinical improvement as defined by IGA for example (i.e. 20 responders versus 20 non-responders), we are powered to see an effect size of 0.66 between the two groups.

Secondary Endpoint: Change in levels of circulating cytokines.

Endpoint Definition: Statistically significant reduction in the cytokine score at 16 weeks of treatment as defined by two-sided, paired Student's t-test.

Method: To determine the impact of Tofacitinib treatment on the Cytokine Score values (a composite Z score derived from the measurements of IP10, TNF-α, CRP, and IL6), a two-sided, paired Student's t-test will be used to compare baseline and 16 weeks of treatment for participants with good compliance with the study drug (2 or fewer missed pills) and without a confounding reported adverse event in the 7 days prior to the Visit 7 endpoint. With 40 participants, we have 80% power to detect an effect size of 0.45 with a p-value less than 0.05, as calculated by G*Power software.

Exploratory Research Goals: For an exploratory component of this analysis to test the impact of Tofacitinib on individual plasma cytokine levels (15+ cytokines measured in total, see *Research Strategy*), using a non-parametric Kolmogorov-Smirnoff (KS) test with 40 participants comparing baseline and 16 weeks of treatment, we have 80% power to detect and effect size of 0.47 with a p-value <0.05, as calculated by G*Power software, using FDR correction for multiple testing on 15 cytokines. This investigation will include cytokines isolated from both the blood and non-invasive skin tapes. For the exploratory goal of defining differences between responders and non-responders, assuming that half of the participants will see clinical improvement, we are powered to see an effect size of 0.66 for the composite Cytokine Score and 0.68 for individual cytokines.

Tertiary Endpoint: Change in levels of neurotoxic metabolites in the kynurenine pathway.

Endpoint Definition: Statistically significant reduction in one or more of three kynurenine dysregulation values at 16 weeks of treatment as defined by a two-sided, paired Student's t-test with FDR correction.

Method: To determine the impact of Tofacitinib treatment on the levels of kynurenine, quinolinic acid, and the kynurenine/tryptophan ratio, a two-sided, paired Student's t-test will be used to compare baseline and 16 weeks of treatment for participants with good compliance with the study drug (2 or fewer missed pills) and without a confounding reported adverse event in the 7 days prior to the Visit 7 endpoint. With 40 participants, we have 80% power to detect an effect size of 0.45 with a p-value less than 0.05, as calculated by G*Power software, and using FDR correction for multiple testing on three KYN pathway dysregulation values.

Exploratory Research Goal: For the exploratory goal of identifying differences between clinical responders and non-responders, assuming that half of the participants will see clinical improvement (i.e. 20 responders versus 20 non-responders), we are powered to see an effect size of 0.66 between the two groups, as calculated by G*Power software.

<u>Tertiary Endpoint:</u> Change in levels of autoantibodies associated with AITD and celiac disease.

Endpoint Definition: Statistically significant reduction in levels of one or more autoantibodies at 16 weeks as defined by a two-sided, paired Student's t-test.

Method: To determine the impact of Tofacitinib on levels of autoantibodies associated with 1) AITD, i.e. anti-thyroperoxidase (TPO), anti-thyroglobulin (TG), and anti-thyroid stimulating hormone receptor (TSHr), and 2) celiac disease, i.e. anti-tissue transglutaminase (TG) and deamidated gliadin peptide (DGP), a two-sided Student's t-test will be used to compare baseline and 16 weeks of treatment. With 40 participants, we have 80% power to detect an effect size of 0.45 with an FDR corrected p-value less than 0.05, as calculated by G*Power software.

<u>Specific Aim 3. To define the impact of JAK inhibition on immune skin conditions in Down syndrome.</u>

<u>Secondary Endpoint:</u> Change in IGA, DLQI, and/or disease-specific scores.

Endpoint Definition: A statistically significant *percentage reduction* in one or more metrics of skin pathology, including: a) IGA values, b) DLQI values, and/or c) disease-specific scores between baseline and 16 weeks as defined by a two-sided, paired Student's t-test.

Method: The first endpoint for this Specific Aim will be to monitor changes in the Investigator's Global Assessment (IGA), which is a well-established, disease-agnostic measurement of skin disease severity that employs a continuous visual analog scale and has good test-retest reliability. A second disease-agnostic secondary endpoint will be the Dermatology Life Quality Index (DLQI), a patient-reported outcome that is commonly used in dermatology trials. The third secondary endpoint for this study is a percentage reduction in the disease-specific measurements used to monitor each skin condition. We will employ Eczema Area and Severity Index (EASI) for AD, Severity of Alopecia Tool (SALT) for AA, Modified Sartorius Score (MSS) for HS, Psoriasis Area and Severity Index (PASI) for psoriasis, and Vitiligo Extent Tensity Index (VETI) for vitiligo. By focusing on a *percentage reduction* in disease-specific scores, we will be able to compare values from participants with different conditions. In every case (IGA, DLQI or disease-specific scores) we will define whether the percentage reduction reaches significance using a two-sided, paired Student's t-test comparing baseline and 16 weeks of treatment. In every case, for 40 individuals, we have 80% power to detect an effect size of 0.45 with a p-value less than 0.05, as calculated by G*Power software. Finally, although we will be analyzing changes between baseline and 16 weeks, data will be also gathered at the intermediate time point of eight weeks for research purposes.

<u>Specific Aim 4. To characterize the impact of JAK inhibition on cognition and quality of life in Down syndrome.</u>

<u>Tertiary Endpoint:</u> Change in any one or more of the cognitive assessments performed.

Endpoint Definition: a statistically significant improvement in any one of the cognitive measures employed between baseline and 16 weeks as defined by a Wilcoxon signed-rank test with FDR correction or a two-sided, paired Student's t-test, as appropriate.

Method: This Aim includes exploratory tertiary endpoints to determine the impact of Tofacitinib treatment on cognitive outcomes in people with DS using cognitive assessments and their subdomains. For example, for Leiter 3 subdomains, we will employ a Wilcoxon signed-rank test (matched pairs) with which we have 80% power to detect an effect size of 0.32 with an FDR-corrected p-value less than 0.05, as calculated by G*Power software. For CANTAB subdomains, we will employ two-sided, paired Student's t-test comparing baseline and 16 weeks of treatment, we have 80% power to detect an effect size of 0.45 with an FDR-corrected p-value less than 0.05, as calculated by G*Power software.

<u>Tertiary Endpoint:</u> Change in PROMIS surveys of quality of life.

Endpoint Definition: A statistically significant improvement in any one or more of the NIH PROMIS tools employed between baseline and 16 weeks as defined by a two-sided, paired Student's t-test.

Method: Efforts in this Aim also include the NIH-developed PROMIS tool for quality of life assessment. Here, we will employ two-sided, paired Student's t-test comparing baseline and 16 weeks of treatment, we have 80% power to detect an effect size of 0.45 with a p-value less than 0.05 as calculated by G*Power software.

<u>Secondary analysis to investigate potential age- and sex-dependent effects.</u>

Although we are unlikely to have sufficient numbers for separate analysis of effects in children versus adults or females versus males, it is possible that the effects of the study drug may differ with age and/or sex, potentially reducing our power to detect significant differences in endpoint measures. To address this issue, we will conduct a series of secondary analyses, which would not affect the definition of the endpoints or the assessment of whether the endpoints were met or not, to investigate potential effects of age and sex as modifiers of the effects of the study drug.

Toward this end, we will use repeated measures ANOVA to test for significant interaction of outcome variables with either age (as a continuous variable) or sex. These analyses will yield p-values and estimates of the difference in effect size per unit of age, or between females and males, for each of the outcome measures. This would allow us to better interpret any potential failure to meet endpoint definitions.

VIII. Summarize Knowledge to be Gained:

The potential knowledge that may be gained from this clinical trial is substantial. The biomedical significance of immune disorders is undisputed – approximately 10% of the American population suffers from an autoimmune disease resulting in hundreds of millions of dollars in medical expenses. Furthermore, individuals with DS are likely the largest human population with a genetic predisposition to autoimmunity and inflammatory disorders, which we hypothesize is due to the consistent activation of the IFN response and consequent widespread immune dysregulation. This clinical trial will, for the first time, evaluate in DS the impact of JAK inhibition on immune skin conditions, on molecular markers of IFN activity and immune dysregulation in DS, and on cognition.

If JAK inhibition proves to beneficial in any of the endpoints in this trial, this knowledge would have the potential to not only advance the understanding of DS as an immune disorder, but also to advance the mechanistic understanding of the impacts of immune dysregulation in general. Furthermore, the clinical trial will provide a multidimensional analysis of diverse yet intertwined biological processes – immune skin conditions, general immune dysregulation, IFN signaling, and cognition – which has the potential to accelerate the repurposing of JAK inhibitors, or other anti-IFN strategies, for the treatment of a wide range of immune and neurological conditions thought to be driven by IFN signaling. For example, we hypothesize that JAK inhibition could provide therapeutic benefits for diverse autoimmune conditions more prevalent in

DS, pulmonary hypertension, cases of developmental regression, various seizure disorders, and even perhaps be used in combination with anti-amyloid strategies for the treatment of Alzheimer's disease in DS.

IX. References:

1. Alexander M, Petri H, Ding Y, Wandel C, Khwaja O, Foskett N. Morbidity and medication in a large population of individuals with Down syndrome compared to the general population. Developmental medicine and child neurology. 2015. doi: 10.1111/dmcn.12868. PubMed PMID: 26282180.
2. Stoll C, Alembik Y, Dott B, Roth MP. Study of Down syndrome in 238,942 consecutive births. Annales de genetique. 1998;41(1):44-51. PubMed PMID: 9599651.
3. Bratman SV, Horst KC, Carlson RW, Kapp DS. Solid malignancies in individuals with Down syndrome: a case presentation and literature review. J Natl Compr Canc Netw. 2014;12(11):1537-45. PubMed PMID: 25361800.
4. Xavier AC, Ge Y, Taub JW. Down syndrome and malignancies: a unique clinical relationship: a paper from the 2008 william beaumont hospital symposium on molecular pathology. J Mol Diagn. 2009;11(5):371-80. doi: 10.2353/jmoldx.2009.080132. PubMed PMID: 19710397; PMCID: PMC2729834.
5. Wiseman FK, Al-Janabi T, Hardy J, Karmiloff-Smith A, Nizetic D, Tybulewicz VL, Fisher EM, Strydom A. A genetic cause of Alzheimer disease: mechanistic insights from Down syndrome. Nat Rev Neurosci. 2015;16(9):564-74. doi: 10.1038/nrn3983. PubMed PMID: 26243569.
6. Loudon MM, Day RE, Duke EM. Thyroid dysfunction in Down's syndrome. Archives of disease in childhood. 1985;60(12):1149-51. Epub 1985/12/01. PubMed PMID: 2936311; PMCID: PMC1777686.
7. Friedman DL, Kastner T, Pond WS, O'Brien DR. Thyroid dysfunction in individuals with Down syndrome. Archives of internal medicine. 1989;149(9):1990-3. Epub 1989/09/01. PubMed PMID: 2528336.
8. Dinani S, Carpenter S. Down's syndrome and thyroid disorder. Journal of mental deficiency research. 1990;34 (Pt 2):187-93. Epub 1990/04/01. PubMed PMID: 2140417.
9. Zori RT, Schatz DA, Ostrer H, Williams CA, Spillar R, Riley WJ. Relationship of autoimmunity to thyroid dysfunction in children and adults with Down syndrome. Am J Med Genet Suppl. 1990;7:238-41. Epub 1990/01/01. PubMed PMID: 2149955.
10. Ivarsson SA, Ericsson UB, Gustafsson J, Forslund M, Vegfors P, Anneren G. The impact of thyroid autoimmunity in children and adolescents with Down syndrome. Acta paediatrica (Oslo, Norway : 1992). 1997;86(10):1065-7. Epub 1997/11/14. PubMed PMID: 9350885.
11. Karlsson B, Gustafsson J, Hedov G, Ivarsson SA, Anneren G. Thyroid dysfunction in Down's syndrome: relation to age and thyroid autoimmunity. Archives of disease in childhood. 1998;79(3):242-5. Epub 1999/01/06. PubMed PMID: 9875020; PMCID: PMC1717691.
12. Chen MH, Chen SJ, Su LY, Yang W. Thyroid dysfunction in patients with Down syndrome. Acta Paediatr Taiwan. 2007;48(4):191-5. Epub 2008/02/13. PubMed PMID: 18265539.
13. Lazzari R, Collina A, Arena G, Corvaglia L, Marzatico M, Vallini M, Bochicchio A, Pasetti A, Frassineti S, Forchielli L. [Celiac disease in children with Down's syndrome]. La Pediatria medica e chirurgica : Medical and surgical pediatrics. 1994;16(5):467-70. Epub 1994/09/01. PubMed PMID: 7885956.
14. George EK, Mearin ML, Bouquet J, von Blomberg BM, Stapel SO, van Elburg RM, de Graaf EA. High frequency of celiac disease in Down syndrome. The Journal of pediatrics. 1996;128(4):555-7. Epub 1996/04/01. PubMed PMID: 8618192.
15. Pueschel SM, Romano C, Failla P, Barone C, Pettinato R, Castellano Chiodo A, Plumari DL. A prevalence study of celiac disease in persons with Down syndrome residing in the United States of America. Acta paediatrica (Oslo, Norway : 1992). 1999;88(9):953-6. Epub 1999/10/16. PubMed PMID: 10519335.
16. Zachor DA, Mroczek-Musulman E, Brown P. Prevalence of celiac disease in Down syndrome in the United States. Journal of pediatric gastroenterology and nutrition. 2000;31(3):275-9. Epub 2000/09/21. PubMed PMID: 10997372.
17. Mackey J, Treem WR, Worley G, Boney A, Hart P, Kishnani PS. Frequency of celiac disease in individuals with Down syndrome in the United States. Clinical pediatrics. 2001;40(5):249-52. Epub 2001/06/05. doi: 10.1177/000992280104000502. PubMed PMID: 11388673; PMCID: PMC2706421.
18. Cogulu O, Ozkinay F, Gunduz C, Cankaya T, Aydogdu S, Ozgenc F, Kutukculer N, Ozkinay C. Celiac disease in children with Down syndrome: importance of follow-up and serologic screening.

Pediatrics international : official journal of the Japan Pediatric Society. 2003;45(4):395-9. Epub 2003/08/13. PubMed PMID: 12911473.
19. Marild K, Stephansson O, Grahnquist L, Cnattingius S, Soderman G, Ludvigsson JF. Down syndrome is associated with elevated risk of celiac disease: a nationwide case-control study. The Journal of pediatrics. 2013;163(1):237-42. doi: 10.1016/j.jpeds.2012.12.087. PubMed PMID: 23399451.
20. Anwar AJ, Walker JD, Frier BM. Type 1 diabetes mellitus and Down's syndrome: prevalence, management and diabetic complications. Diabetic medicine : a journal of the British Diabetic Association. 1998;15(2):160-3. doi: 10.1002/(SICI)1096-9136(199802)15:2<160::AID-DIA537>3.0.CO;2-J. PubMed PMID: 9507919.
21. Bergholdt R, Eising S, Nerup J, Pociot F. Increased prevalence of Down's syndrome in individuals with type 1 diabetes in Denmark: A nationwide population-based study. Diabetologia. 2006;49(6):1179-82. doi: 10.1007/s00125-006-0231-6. PubMed PMID: 16575558.
22. Rohrer TR, Hennes P, Thon A, Dost A, Grabert M, Rami B, Wiegand S, Holl RW, Initiative DPV. Down's syndrome in diabetic patients aged <20 years: an analysis of metabolic status, glycaemic control and autoimmunity in comparison with type 1 diabetes. Diabetologia. 2010;53(6):1070-5. doi: 10.1007/s00125-010-1686-z. PubMed PMID: 20186386.
23. Madan V, Williams J, Lear JT. Dermatological manifestations of Down's syndrome. Clinical and experimental dermatology. 2006;31(5):623-9. doi: 10.1111/j.1365-2230.2006.02164.x. PubMed PMID: 16901300.
24. Sureshbabu R, Kumari R, Ranugha S, Sathyamoorthy R, Udayashankar C, Oudeacoumar P. Phenotypic and dermatological manifestations in Down Syndrome. Dermatology online journal. 2011;17(2):3. PubMed PMID: 21382286.
25. Schepis C, Barone C, Siragusa M, Pettinato R, Romano C. An updated survey on skin conditions in Down syndrome. Dermatology. 2002;205(3):234-8. doi: 65859. PubMed PMID: 12399669.
26. Marmon S, De Souza A, Strober BE. Psoriasis and Down syndrome: a report of three cases and a potential pathophysiologic link. Dermatology online journal. 2012;18(6):13. Epub 2012/07/04. PubMed PMID: 22747937.
27. Sullivan KD, Lewis HC, Hill AA, Pandey A, Jackson LP, Cabral JM, Smith KP, Liggett LA, Gomez EB, Galbraith MD, DeGregori J, Espinosa JM. Trisomy 21 consistently activates the interferon response. Elife. 2016;5. doi: 10.7554/eLife.16220. PubMed PMID: 27472900.
28. Sullivan KD, Evans D, Pandey A, Hraha TH, Smith KP, Markham N, Rachubinski AL, Wolter-Warmerdam K, Hickey F, Espinosa JM, Blumenthal T. Trisomy 21 causes changes in the circulating proteome indicative of chronic autoinflammation. Scientific reports. 2017;7(1):14818. Epub 2017/11/03. doi: 10.1038/s41598-017-13858-3. PubMed PMID: 29093484; PMCID: PMC5665944.
29. Powers RK, Culp-Hill R, Ludwig MP, Smith KP, Waugh KA, Minter R, Tuttle KD, Lewis HC, Rachubinski AL, Granrath RE, Carmona-Iragui M, Wilkerson RB, Kahn DE, Joshi M, Lleo A, Blesa R, Fortea J, D'Alessandro A, Costello JC, Sullivan KD, Espinosa JM. Trisomy 21 activates the kynurenine pathway via increased dosage of interferon receptors. Nat Commun. 2019;10(1):4766. Epub 2019/10/20. doi: 10.1038/s41467-019-12739-9. PubMed PMID: 31628327; PMCID: PMC6800452.
30. Greth W, Robbie GJ, Brohawn P, Hultquist M, Yao B. Targeting the interferon pathway with sifalimumab for the treatment of systemic lupus erythematosus. Immunotherapy. 2017;9(1):57-70. Epub 2016/12/22. doi: 10.2217/imt-2016-0090. PubMed PMID: 28000522.
31. Fritz-French C, Shawahna R, Ward JE, Maroun LE, Tyor WR. The recombinant vaccinia virus gene product, B18R, neutralizes interferon alpha and alleviates histopathological complications in an HIV encephalitis mouse model. Journal of interferon & cytokine research : the official journal of the International Society for Interferon and Cytokine Research. 2014;34(7):510-7. doi: 10.1089/jir.2013.0072. PubMed PMID: 24564363; PMCID: 4080846.
32. Alcami A, Smith GL. The vaccinia virus soluble interferon-gamma receptor is a homodimer. The Journal of general virology. 2002;83(Pt 3):545-9. doi: 10.1099/0022-1317-83-3-545. PubMed PMID: 11842249.

33. Alcami A, Symons JA, Smith GL. The vaccinia virus soluble alpha/beta interferon (IFN) receptor binds to the cell surface and protects cells from the antiviral effects of IFN. Journal of virology. 2000;74(23):11230-9. PubMed PMID: 11070021; PMCID: 113220.
34. Ducreux J, Houssiau FA, Vandepapeliere P, Jorgensen C, Lazaro E, Spertini F, Colaone F, Roucairol C, Laborie M, Croughs T, Grouard-Vogel G, Lauwerys BR. Interferon alpha kinoid induces neutralizing anti-interferon alpha antibodies that decrease the expression of interferon-induced and B cell activation associated transcripts: analysis of extended follow-up data from the interferon alpha kinoid phase I/II study. Rheumatology. 2016;55(10):1901-5. Epub 2016/06/30. doi: 10.1093/rheumatology/kew262. PubMed PMID: 27354683; PMCID: PMC5034220.
35. Damsky W, King BA. JAK inhibitors in dermatology: The promise of a new drug class. Journal of the American Academy of Dermatology. 2017;76(4):736-44. Epub 2017/02/01. doi: 10.1016/j.jaad.2016.12.005. PubMed PMID: 28139263.
36. Schwartz DM, Bonelli M, Gadina M, O'Shea JJ. Type I/II cytokines, JAKs, and new strategies for treating autoimmune diseases. Nature reviews Rheumatology. 2016;12(1):25-36. doi: 10.1038/nrrheum.2015.167. PubMed PMID: 26633291; PMCID: 4688091.
37. Roskoski R, Jr. Janus kinase (JAK) inhibitors in the treatment of inflammatory and neoplastic diseases. Pharmacological research. 2016;111:784-803. doi: 10.1016/j.phrs.2016.07.038. PubMed PMID: 27473820.
38. Jabbari A, Nguyen N, Cerise JE, Ulerio G, de Jong A, Clynes R, Christiano AM, Mackay-Wiggan J. Treatment of an alopecia areata patient with tofacitinib results in regrowth of hair and changes in serum and skin biomarkers. Experimental dermatology. 2016;25(8):642-3. doi: 10.1111/exd.13060. PubMed PMID: 27119625; PMCID: 4963264.
39. Liu LY, Craiglow BG, Dai F, King BA. Tofacitinib for the treatment of severe alopecia areata and variants: A study of 90 patients. Journal of the American Academy of Dermatology. 2017;76(1):22-8. Epub 2016/11/07. doi: 10.1016/j.jaad.2016.09.007. PubMed PMID: 27816293.
40. Bissonnette R, Papp KA, Poulin Y, Gooderham M, Raman M, Mallbris L, Wang C, Purohit V, Mamolo C, Papacharalambous J, Ports WC. Topical tofacitinib for atopic dermatitis: a phase IIa randomized trial. Br J Dermatol. 2016;175(5):902-11. Epub 2016/10/30. doi: 10.1111/bjd.14871. PubMed PMID: 27423107.
41. Papp KA, Menter MA, Abe M, Elewski B, Feldman SR, Gottlieb AB, Langley R, Luger T, Thaci D, Buonanno M, Gupta P, Proulx J, Lan S, Wolk R, Pivotal OPT, investigators OPTP. Tofacitinib, an oral Janus kinase inhibitor, for the treatment of chronic plaque psoriasis: results from two randomized, placebo-controlled, phase III trials. Br J Dermatol. 2015;173(4):949-61. Epub 2015/07/08. doi: 10.1111/bjd.14018. PubMed PMID: 26149717.
42. Levy LL, Urban J, King BA. Treatment of recalcitrant atopic dermatitis with the oral Janus kinase inhibitor tofacitinib citrate. Journal of the American Academy of Dermatology. 2015;73(3):395-9. Epub 2015/07/22. doi: 10.1016/j.jaad.2015.06.045. PubMed PMID: 26194706.
43. Craiglow BG, King BA. Tofacitinib Citrate for the Treatment of Vitiligo: A Pathogenesis-Directed Therapy. JAMA Dermatol. 2015;151(10):1110-2. Epub 2015/06/25. doi: 10.1001/jamadermatol.2015.1520. PubMed PMID: 26107994.
44. Bachelez H, van de Kerkhof PC, Strohal R, Kubanov A, Valenzuela F, Lee JH, Yakusevich V, Chimenti S, Papacharalambous J, Proulx J, Gupta P, Tan H, Tawadrous M, Valdez H, Wolk R, Investigators OPTC. Tofacitinib versus etanercept or placebo in moderate-to-severe chronic plaque psoriasis: a phase 3 randomised non-inferiority trial. Lancet. 2015;386(9993):552-61. Epub 2015/06/09. doi: 10.1016/S0140-6736(14)62113-9. PubMed PMID: 26051365.
45. Jabbari A, Dai Z, Xing L, Cerise JE, Ramot Y, Berkun Y, Sanchez GA, Goldbach-Mansky R, Christiano AM, Clynes R, Zlotogorski A. Reversal of Alopecia Areata Following Treatment With the JAK1/2 Inhibitor Baricitinib. EBioMedicine. 2015;2(4):351-5. doi: 10.1016/j.ebiom.2015.02.015. PubMed PMID: 26137574; PMCID: 4486197.
46. Bissonnette R, Luchi M, Fidelus-Gort R, Jackson S, Zhang H, Flores R, Newton R, Scherle P, Yeleswaram S, Chen X, Menter A. A randomized, double-blind, placebo-controlled, dose-escalation study of the safety and efficacy of INCB039110, an oral janus kinase 1 inhibitor, in patients with stable, chronic

plaque psoriasis. J Dermatolog Treat. 2016;27(4):332-8. Epub 2016/01/16. doi: 10.3109/09546634.2015.1115819. PubMed PMID: 26769332.
47. Papp KA, Menter MA, Raman M, Disch D, Schlichting DE, Gaich C, Macias W, Zhang X, Janes JM. A randomized phase 2b trial of baricitinib, an oral Janus kinase (JAK) 1/JAK2 inhibitor, in patients with moderate-to-severe psoriasis. Br J Dermatol. 2016;174(6):1266-76. Epub 2016/01/23. doi: 10.1111/bjd.14403. PubMed PMID: 26800231.
48. FDA grants Fast Track Designation to Aclaris Therapeutics' Investigational JAK Inhibitor for the treatment of Alopecia Areata [Internet]. Globe Newswire. 2018 [cited July 09, 2018]. Available from: https://globenewswire.com/news-release/2018/07/09/1534506/0/en/FDA-grants-Fast-Track-Designation-to-Aclaris-Therapeutics-Investigational-JAK-Inhibitor-for-the-treatment-of-Alopecia-Areata.html.
49. Pfizer Receives Breakthrough Therapy Designation from FDA for PF-06651600, an oral JAK3 Inhibitor, for the Treatment of Patients with Alopecia Areata [Internet]. Business Wire. 2018 [cited September 05, 2018]. Available from: https://www.businesswire.com/news/home/20180905005542/en/Pfizer-Receives-Breakthrough-Therapy-Designation-FDA-PF-06651600.
50. FDA Grants Fast Track Designation to Concert Pharmaceuticals' CTP-543 for the Treatment of Alopecia Areata [Internet]. Business Wire. 2018 [cited January 12, 2018]. Available from: https://www.businesswire.com/news/home/20180112005098/en/FDA-Grants-Fast-Track-Designation-Concert-Pharmaceuticals%E2%80%99.
51. Pfizer Receives Breakthrough Therapy Designation from FDA for PF-04965842, an oral JAK1 Inhibitor, for the Treatment of Patients with Moderate-to-Severe Atopic Dermatitis [Internet]. Business Wire. 2018 [cited February 14, 2018]. Available from: https://www.businesswire.com/news/home/20180214005745/en/Pfizer-Receives-Breakthrough-Therapy-Designation-FDA-PF-04965842.
52. Hansson T, Dahlbom I, Rogberg S, Nyberg BI, Dahlstrom J, Anneren G, Klareskog L, Dannaeus A. Antitissue transglutaminase and antithyroid autoantibodies in children with Down syndrome and celiac disease. Journal of pediatric gastroenterology and nutrition. 2005;40(2):170-4; discussion 25-7. Epub 2005/02/09. PubMed PMID: 15699691.
53. Ji NY, Capone GT, Kaufmann WE. Autism spectrum disorder in Down syndrome: cluster analysis of Aberrant Behaviour Checklist data supports diagnosis. J Intellect Disabil Res. 2011;55(11):1064-77. doi: 10.1111/j.1365-2788.2011.01465.x. PubMed PMID: 21883598.
54. Lim CK, Essa MM, de Paula Martins R, Lovejoy DB, Bilgin AA, Waly MI, Al-Farsi YM, Al-Sharbati M, Al-Shaffae MA, Guillemin GJ. Altered kynurenine pathway metabolism in autism: Implication for immune-induced glutamatergic activity. Autism Res. 2016;9(6):621-31. Epub 2015/10/27. doi: 10.1002/aur.1565. PubMed PMID: 26497015.
55. Ohja K, Gozal E, Fahnestock M, Cai L, Cai J, Freedman JH, Switala A, El-Baz A, Barnes GN. Neuroimmunologic and Neurotrophic Interactions in Autism Spectrum Disorders: Relationship to Neuroinflammation. Neuromolecular Med. 2018;20(2):161-73. Epub 2018/04/25. doi: 10.1007/s12017-018-8488-8. PubMed PMID: 29691724; PMCID: PMC5942347.
56. Johannsen P, Christensen JE, Goldstein H, Nielsen VK, Mai J. Epilepsy in Down syndrome--prevalence in three age groups. Seizure. 1996;5(2):121-5. PubMed PMID: 8795127.
57. Akiyama H. Inflammatory response in Alzheimer's disease. Tohoku J Exp Med. 1994;174(3):295-303. doi: 10.1620/tjem.174.295.
58. Nilsson L, Rogers J, Potter H. The essential role of inflammation and induced gene expression in the pathogenic pathway of Alzheimer's disease. Front Biosci. 1998;3:d436-46. PubMed PMID: 9545438.
59. Akiyama H, Barger S, Barnum S, Bradt B, Bauer J, Cole GM, Cooper NR, Eikelenboom P, Emmerling M, Fiebich BL, Finch CE, Frautschy S, Griffin WS, Hampel H, Hull M, Landreth G, Lue L, Mrak R, Mackenzie IR, McGeer PL, O'Banion MK, Pachter J, Pasinetti G, Plata-Salaman C, Rogers J, Rydel R, Shen Y, Streit W, Strohmeyer R, Tooyoma I, Van Muiswinkel FL, Veerhuis R, Walker D, Webster S, Wegrzyniak B, Wenk G, Wyss-Coray T. Inflammation and Alzheimer's disease. Neurobiology of aging. 2000;21(3):383-421. Epub 2000/06/20. PubMed PMID: 10858586; PMCID: PMC3887148.

60. McGeer PL, Rogers J, McGeer EG. Inflammation, anti-inflammatory agents and Alzheimer disease: the last 12 years. J Alzheimers Dis. 2006;9(3 Suppl):271-6. PubMed PMID: 16914866.
61. Holmes C, Cunningham C, Zotova E, Woolford J, Dean C, Kerr S, Culliford D, Perry VH. Systemic inflammation and disease progression in Alzheimer disease. Neurology. 2009;73(10):768-74. doi: 73/10/768 [pii] 10.1212/WNL.0b013e3181b6bb95.
62. Swardfager W, Lanctot K, Rothenburg L, Wong A, Cappell J, Herrmann N. A meta-analysis of cytokines in Alzheimer's disease. Biol Psychiatry. 2010;68(10):930-41. Epub 2010/08/10. doi: 10.1016/j.biopsych.2010.06.012. PubMed PMID: 20692646.
63. Leung R, Proitsi P, Simmons A, Lunnon K, Guntert A, Kronenberg D, Pritchard M, Tsolaki M, Mecocci P, Kloszewska I, Vellas B, Soininen H, Wahlund LO, Lovestone S. Inflammatory proteins in plasma are associated with severity of Alzheimer's disease. PloS one. 2013;8(6):e64971. doi: 10.1371/journal.pone.0064971. PubMed PMID: 23762274; PMCID: 3677891.
64. Sudduth TL, Schmitt FA, Nelson PT, Wilcock DM. Neuroinflammatory phenotype in early Alzheimer's disease. Neurobiology of aging. 2013;34(4):1051-9. Epub 2012/10/16. doi: 10.1016/j.neurobiolaging.2012.09.012. PubMed PMID: 23062700; PMCID: PMC3579221.
65. Islam N, Leung PS, Huntley AC, Gershwin ME. The autoimmune basis of alopecia areata: a comprehensive review. Autoimmunity reviews. 2015;14(2):81-9. Epub 2014/10/16. doi: 10.1016/j.autrev.2014.10.014. PubMed PMID: 25315746.
66. Xing L, Dai Z, Jabbari A, Cerise JE, Higgins CA, Gong W, de Jong A, Harel S, DeStefano GM, Rothman L, Singh P, Petukhova L, Mackay-Wiggan J, Christiano AM, Clynes R. Alopecia areata is driven by cytotoxic T lymphocytes and is reversed by JAK inhibition. Nature medicine. 2014;20(9):1043-9. doi: 10.1038/nm.3645. PubMed PMID: 25129481; PMCID: 4362521.
67. Harris JE, Rashighi M, Nguyen N, Jabbari A, Ulerio G, Clynes R, Christiano AM, Mackay-Wiggan J. Rapid skin repigmentation on oral ruxolitinib in a patient with coexistent vitiligo and alopecia areata (AA). Journal of the American Academy of Dermatology. 2016;74(2):370-1. doi: 10.1016/j.jaad.2015.09.073. PubMed PMID: 26685721; PMCID: 4718770.
68. Mackay-Wiggan J, Jabbari A, Nguyen N, Cerise JE, Clark C, Ulerio G, Furniss M, Vaughan R, Christiano AM, Clynes R. Oral ruxolitinib induces hair regrowth in patients with moderate-to-severe alopecia areata. JCI insight. 2016;1(15):e89790. doi: 10.1172/jci.insight.89790. PubMed PMID: 27699253; PMCID: 5033756.
69. Sanchez GAM, Reinhardt A, Ramsey S, Wittkowski H, Hashkes PJ, Berkun Y, Schalm S, Murias S, Dare JA, Brown D, Stone DL, Gao L, Klausmeier T, Foell D, Jesus AA, Chapelle DC, Kim H, Dill S, Colbert R, Failla L, Kost B, O'Brien M, Reynolds JC, Folio LR, Calvo KR, Paul SM, Weir N, Brofferio A, Soldatos A, Biancotto A, Cowen EW, Digiovanna JG, Gadina M, Lipton AJ, Hadigan C, Holland SM, Fontana J, Alawad AS, Brown RJ, Rother KI, Heller T, Brooks KM, Kumar P, Brooks SR, Waldman M, Singh HK, Nickeleit V, Silk M, Prakash A, Janes JM, Ozen S, Wakim PG, Brogan PA, Macias WL, Goldbach-Mansky R. JAK1/2 inhibition with baricitinib in the treatment of autoinflammatory interferonopathies. The Journal of clinical investigation. 2018. Epub 2018/04/13. doi: 10.1172/JCI98814. PubMed PMID: 29649002.
70. Assassi S, Mayes MD, Arnett FC, Gourh P, Agarwal SK, McNearney TA, Chaussabel D, Oommen N, Fischbach M, Shah KR, Charles J, Pascual V, Reveille JD, Tan FK. Systemic sclerosis and lupus: points in an interferon-mediated continuum. Arthritis Rheum. 2010;62(2):589-98. Epub 2010/01/30. doi: 10.1002/art.27224. PubMed PMID: 20112391; PMCID: PMC2879587.
71. Baechler EC, Batliwalla FM, Karypis G, Gaffney PM, Ortmann WA, Espe KJ, Shark KB, Grande WJ, Hughes KM, Kapur V, Gregersen PK, Behrens TW. Interferon-inducible gene expression signature in peripheral blood cells of patients with severe lupus. Proc Natl Acad Sci U S A. 2003;100(5):2610-5. Epub 2003/02/27. doi: 10.1073/pnas.0337679100. PubMed PMID: 12604793; PMCID: PMC151388.
72. Hassanain HH, Chon SY, Gupta SL. Differential regulation of human indoleamine 2,3-dioxygenase gene expression by interferons-gamma and -alpha. Analysis of the regulatory region of the gene and identification of an interferon-gamma-inducible DNA-binding factor. J Biol Chem. 1993;268(7):5077-84. Epub 1993/03/05. PubMed PMID: 8444884.
73. Guillemin GJ. Quinolinic acid, the inescapable neurotoxin. The FEBS journal. 2012;279(8):1356-65. Epub 2012/01/18. doi: 10.1111/j.1742-4658.2012.08485.x. PubMed PMID: 22248144.

74. Keilhoff G, Wolf G. Memantine prevents quinolinic acid-induced hippocampal damage. Eur J Pharmacol. 1992;219(3):451-4. Epub 1992/09/04. PubMed PMID: 1425971.
75. Costa AC, Scott-McKean JJ, Stasko MR. Acute injections of the NMDA receptor antagonist memantine rescue performance deficits of the Ts65Dn mouse model of Down syndrome on a fear conditioning test. Neuropsychopharmacology. 2008;33(7):1624-32. Epub 2007/08/19. doi: 10.1038/sj.npp.1301535. PubMed PMID: 17700645.
76. Lockrow J, Boger H, Bimonte-Nelson H, Granholm AC. Effects of long-term memantine on memory and neuropathology in Ts65Dn mice, a model for Down syndrome. Behav Brain Res. 2011;221(2):610-22. doi: 10.1016/j.bbr.2010.03.036. PubMed PMID: 20363261; PMCID: PMC2928411.
77. Ahmed MM, Dhanasekaran AR, Block A, Tong S, Costa AC, Gardiner KJ. Protein profiles associated with context fear conditioning and their modulation by memantine. Mol Cell Proteomics. 2014;13(4):919-37. doi: 10.1074/mcp.M113.035568. PubMed PMID: 24469516; PMCID: PMC3977192.
78. Braidy N, Grant R. Kynurenine pathway metabolism and neuroinflammatory disease. Neural Regen Res. 2017;12(1):39-42. Epub 2017/03/03. doi: 10.4103/1673-5374.198971. PubMed PMID: 28250737; PMCID: PMC5319230.
79. Breda C, Sathyasaikumar KV, Sograte Idrissi S, Notarangelo FM, Estranero JG, Moore GG, Green EW, Kyriacou CP, Schwarcz R, Giorgini F. Tryptophan-2,3-dioxygenase (TDO) inhibition ameliorates neurodegeneration by modulation of kynurenine pathway metabolites. Proc Natl Acad Sci U S A. 2016;113(19):5435-40. Epub 2016/04/27. doi: 10.1073/pnas.1604453113. PubMed PMID: 27114543; PMCID: PMC4868470.
80. Davis I, Liu A. What is the tryptophan kynurenine pathway and why is it important to neurotherapeutics? Expert Rev Neurother. 2015;15(7):719-21. Epub 2015/05/26. doi: 10.1586/14737175.2015.1049999. PubMed PMID: 26004930; PMCID: PMC4482796.
81. Gulaj E, Pawlak K, Bien B, Pawlak D. Kynurenine and its metabolites in Alzheimer's disease patients. Adv Med Sci. 2010;55(2):204-11. Epub 2010/07/20. doi: 10.2478/v10039-010-0023-6. PubMed PMID: 20639188.
82. Lovelace MD, Varney B, Sundaram G, Lennon MJ, Lim CK, Jacobs K, Guillemin GJ, Brew BJ. Recent evidence for an expanded role of the kynurenine pathway of tryptophan metabolism in neurological diseases. Neuropharmacology. 2017;112(Pt B):373-88. Epub 2016/03/21. doi: 10.1016/j.neuropharm.2016.03.024. PubMed PMID: 26995730.
83. Savitz J. Role of Kynurenine Metabolism Pathway Activation in Major Depressive Disorders. Curr Top Behav Neurosci. 2017;31:249-67. Epub 2016/05/26. doi: 10.1007/7854_2016_12. PubMed PMID: 27221627.
84. Schwarcz R, Stone TW. The kynurenine pathway and the brain: Challenges, controversies and promises. Neuropharmacology. 2017;112(Pt B):237-47. Epub 2016/08/12. doi: 10.1016/j.neuropharm.2016.08.003. PubMed PMID: 27511838.
85. Numminen H, Service E, Ahonen T, Ruoppila I. Working memory and everyday cognition in adults with Down's syndrome. Journal of Intellectual Disability Research. 2001;45:157-68. doi: 10.1046/j.1365-2788.2001.00298.x. PubMed PMID: WOS:000168261000010.
86. Silverman W. Down syndrome: Cognitive phenotype. Mental Retardation and Developmental Disabilities Research Reviews. 2007;13(3):228-36. doi: 10.1002/mrdd.20156. PubMed PMID: WOS:000250194000006.
87. Hoftberger R, Garzuly F, Dienes HP, Grubits J, Rohonyi B, Fischer G, Hanzely Z, Lassmann H, Budka H. Fulminant central nervous system demyelination associated with interferon-alpha therapy and hepatitis C virus infection. Mult Scler. 2007;13(9):1100-6. Epub 2007/10/31. doi: 10.1177/1352458507078684. PubMed PMID: 17967838.
88. Wang J, Lin W, Popko B, Campbell IL. Inducible production of interferon-gamma in the developing brain causes cerebellar dysplasia with activation of the Sonic hedgehog pathway. Mol Cell Neurosci. 2004;27(4):489-96. Epub 2004/11/24. doi: 10.1016/j.mcn.2004.08.004. PubMed PMID: 15555926.
89. Edgin JO. Cognition in Down syndrome: a developmental cognitive neuroscience perspective. Wiley Interdiscip Rev Cogn Sci. 2013;4(3):307-17. Epub 2013/05/01. doi: 10.1002/wcs.1221. PubMed PMID: 26304208.

90. Wilcock DM. Neuroinflammation in the aging down syndrome brain; lessons from Alzheimer's disease. Current gerontology and geriatrics research. 2012;2012:170276. doi: 10.1155/2012/170276 [doi].
91. Wilcock DM, Griffin WS. Down's syndrome, neuroinflammation, and Alzheimer neuropathogenesis. J Neuroinflammation. 2013;10:84. doi: 10.1186/1742-2094-10-84. PubMed PMID: 23866266; PMCID: PMC3750399.
92. Iyer AM, van Scheppingen J, Milenkovic I, Anink JJ, Adle-Biassette H, Kovacs GG, Aronica E. mTOR Hyperactivation in down syndrome hippocampus appears early during development. J Neuropathol Exp Neurol. 2014;73(7):671-83. doi: 10.1097/NEN.0000000000000083. PubMed PMID: 24918639.
93. Troca-Marin JA, Casanas JJ, Benito I, Montesinos ML. The Akt-mTOR pathway in Down's syndrome: the potential use of rapamycin/rapalogs for treating cognitive deficits. CNS Neurol Disord Drug Targets. 2014;13(1):34-40. PubMed PMID: 24152334.
94. Di Domenico F, Tramutola A, Foppoli C, Head E, Perluigi M, Butterfield DA. mTOR in Down syndrome: Role in Ass and tau neuropathology and transition to Alzheimer disease-like dementia. Free Radic Biol Med. 2018;114:94-101. Epub 2017/08/16. doi: 10.1016/j.freeradbiomed.2017.08.009. PubMed PMID: 28807816; PMCID: PMC5748251.
95. Perluigi M, Pupo G, Tramutola A, Cini C, Coccia R, Barone E, Head E, Butterfield DA, Di Domenico F. Neuropathological role of PI3K/Akt/mTOR axis in Down syndrome brain. Biochim Biophys Acta. 2014;1842(7):1144-53. doi: 10.1016/j.bbadis.2014.04.007. PubMed PMID: 24735980; PMCID: PMC4062876.
96. Wilcock DM, Hurban J, Helman AM, Sudduth TL, McCarty KL, Beckett TL, Ferrell JC, Murphy MP, Abner EL, Schmitt FA, Head E. Down syndrome individuals with Alzheimer's disease have a distinct neuroinflammatory phenotype compared to sporadic Alzheimer's disease. Neurobiology of aging. 2015;36(9):2468-74. doi: 10.1016/j.neurobiolaging.2015.05.016. PubMed PMID: 26103884; PMCID: 4602365.
97. Head E, Lott IT, Wilcock DM, Lemere CA. Aging in Down Syndrome and the Development of Alzheimer's Disease Neuropathology. Curr Alzheimer Res. 2016;13(1):18-29. PubMed PMID: 26651341; PMCID: PMC4948181.
98. Wilcock DM, Schmitt FA, Head E. Cerebrovascular contributions to aging and Alzheimer's disease in Down syndrome. Biochim Biophys Acta. 2016;1862(5):909-14. doi: 10.1016/j.bbadis.2015.11.007. PubMed PMID: 26593849; PMCID: PMC4821721.
99. Dipasquale O, Cooper EA, Tibble J, Voon V, Baglio F, Baselli G, Cercignani M, Harrison NA. Interferon-alpha acutely impairs whole-brain functional connectivity network architecture - A preliminary study. Brain Behavior and Immunity. 2016;58:31-9. doi: 10.1016/j.bbi.2015.12.011. PubMed PMID: WOS:000385905600005.
100. Minter MR, Moore Z, Zhang M, Brody KM, Jones NC, Shultz SR, Taylor JM, Crack PJ. Deletion of the type-1 interferon receptor in APPSWE/PS1DeltaE9 mice preserves cognitive function and alters glial phenotype. Acta neuropathologica communications. 2016;4(1):72. doi: 10.1186/s40478-016-0341-4. PubMed PMID: 27400725; PMCID: 4940712.
101. Lucaciu LA, Dumitrascu DL. Depression and suicide ideation in chronic hepatitis C patients untreated and treated with interferon: prevalence, prevention, and treatment. Annals of gastroenterology : quarterly publication of the Hellenic Society of Gastroenterology. 2015;28(4):440-7. PubMed PMID: 26424594; PMCID: 4585389.
102. Taylor JM, Minter MR, Newman AG, Zhang M, Adlard PA, Crack PJ. Type-1 interferon signaling mediates neuro-inflammatory events in models of Alzheimer's disease. Neurobiology of aging. 2014;35(5):1012-23. doi: 10.1016/j.neurobiolaging.2013.10.089. PubMed PMID: 24262201.
103. Mastrangelo MA, Sudol KL, Narrow WC, Bowers WJ. Interferon-{gamma} differentially affects Alzheimer's disease pathologies and induces neurogenesis in triple transgenic-AD mice. Am J Pathol. 2009;175(5):2076-88. doi: 10.2353/ajpath.2009.090059. PubMed PMID: 19808651; PMCID: PMC2774071.
104. Wichers MC, Koek GH, Robaeys G, Verkerk R, Scharpe S, Maes M. IDO and interferon-alpha-induced depressive symptoms: a shift in hypothesis from tryptophan depletion to neurotoxicity. Mol Psychiatry. 2005;10(6):538-44. Epub 2004/10/21. doi: 10.1038/sj.mp.4001600. PubMed PMID: 15494706.

105. Maroun LE. Interferon action and chromosome 21 trisomy (Down syndrome): 15 years later. Journal of theoretical biology. 1996;181(1):41-6. doi: 10.1006/jtbi.1996.0113. PubMed PMID: 8796190.
106. Bialas AR, Presumey J, Das A, van der Poel CE, Lapchak PH, Mesin L, Victora G, Tsokos GC, Mawrin C, Herbst R, Carroll MC. Microglia-dependent synapse loss in type I interferon-mediated lupus. Nature. 2017;546(7659):539-43. Epub 2017/06/15. doi: 10.1038/nature22821. PubMed PMID: 28614301.
107. Fukuyama T, Tschernig T, Qi Y, Volmer DA, Baumer W. Aggression behaviour induced by oral administration of the Janus-kinase inhibitor tofacitinib, but not oclacitinib, under stressful conditions. Eur J Pharmacol. 2015;764:278-82. Epub 2015/07/15. doi: 10.1016/j.ejphar.2015.06.060. PubMed PMID: 26164790.
108. Schwartz DM, Kanno Y, Villarino A, Ward M, Gadina M, O'Shea JJ. JAK inhibition as a therapeutic strategy for immune and inflammatory diseases. Nature reviews Drug discovery. 2017;17(1):78. Epub 2017/12/29. doi: 10.1038/nrd.2017.267. PubMed PMID: 29282366; PMCID: PMC6168198.
109. Cohen SB, Tanaka Y, Mariette X, Curtis JR, Lee EB, Nash P, Winthrop KL, Charles-Schoeman C, Thirunavukkarasu K, DeMasi R, Geier J, Kwok K, Wang L, Riese R, Wollenhaupt J. Long-term safety of tofacitinib for the treatment of rheumatoid arthritis up to 8.5 years: integrated analysis of data from the global clinical trials. Ann Rheum Dis. 2017;76(7):1253-62. Epub 2017/02/02. doi: 10.1136/annrheumdis-2016-210457. PubMed PMID: 28143815; PMCID: PMC5530353.
110. Powers RK, Sullivan, K.D., Culp-Hill, R., Ludwig, M..P, Smith, K..P, Waugh, K.A., Minter, R., Tuttle, K.D., Lewis, H.C., Rachubinski, A.L., Granrath, R.E., Wilkerson, R.B. Kahn, D.E., Joshi, M., D'Alessandro, A., Costello, J.C., and Espinosa, J.M. Trisomy 21 activates the kynurenine pathway via increased dosage of interferon receptors
. BioRxiv (under review in Nature Communications)2019.
111. Anders S, Pyl PT, Huber W. HTSeq--a Python framework to work with high-throughput sequencing data. Bioinformatics. 2015;31(2):166-9. doi: 10.1093/bioinformatics/btu638. PubMed PMID: 25260700; PMCID: 4287950.
112. D'Alessandro A, Gevi F, Zolla L. Targeted mass spectrometry-based metabolomic profiling through multiple reaction monitoring of liver and other biological matrices. Methods Mol Biol. 2012;909:279-94. Epub 2012/08/21. doi: 10.1007/978-1-61779-959-4_18. PubMed PMID: 22903722.
113. Gehrke S, Reisz JA, Nemkov T, Hansen KC, D'Alessandro A. Characterization of rapid extraction protocols for high-throughput metabolomics. Rapid Commun Mass Spectrom. 2017;31(17):1445-52. Epub 2017/06/07. doi: 10.1002/rcm.7916. PubMed PMID: 28586533; PMCID: PMC5547002.
114. Wouters J, Weijerman ME, van Furth AM, Schreurs MW, Crusius JB, von Blomberg BM, de Baaij LR, Broers CJ, Gemke RJ. Prospective human leukocyte antigen, endomysium immunoglobulin A antibodies, and transglutaminase antibodies testing for celiac disease in children with Down syndrome. The Journal of pediatrics. 2009;154(2):239-42. Epub 2008/09/30. doi: 10.1016/j.jpeds.2008.08.007. PubMed PMID: 18822429.
115. Carlsson A, Axelsson I, Borulf S, Bredberg A, Forslund M, Lindberg B, Sjoberg K, Ivarsson SA. Prevalence of IgA-antigliadin antibodies and IgA-antiendomysium antibodies related to celiac disease in children with Down syndrome. Pediatrics. 1998;101(2):272-5. Epub 1998/01/31. PubMed PMID: 9445503.
116. Bilgili SG, Akdeniz N, Karadag AS, Akbayram S, Calka O, Ozkol HU. Mucocutaneous disorders in children with down syndrome: case-controlled study. Genetic counseling. 2011;22(4):385-92. PubMed PMID: 22303799.
117. Daneshpazhooh M, Nazemi TM, Bigdeloo L, Yoosefi M. Mucocutaneous findings in 100 children with Down syndrome. Pediatric dermatology. 2007;24(3):317-20. doi: 10.1111/j.1525-1470.2007.00412.x. PubMed PMID: 17542890.
118. Sago H, Carlson EJ, Smith DJ, Kilbridge J, Rubin EM, Mobley WC, Epstein CJ, Huang TT. Ts1Cje, a partial trisomy 16 mouse model for Down syndrome, exhibits learning and behavioral abnormalities. Proc Natl Acad Sci U S A. 1998;95(11):6256-61. Epub 1998/05/30. PubMed PMID: 9600952; PMCID: PMC27649.
119. Finlay AY, Basra MKA, Piguet V, Salek MS. Dermatology life quality index (DLQI): a paradigm shift to patient-centered outcomes. J Invest Dermatol. 2012;132(10):2464-5. Epub 2012/05/18. doi: 10.1038/jid.2012.147. PubMed PMID: 22592153.

120. Eichenfield LF, Tom WL, Chamlin SL, Feldman SR, Hanifin JM, Simpson EL, Berger TG, Bergman JN, Cohen DE, Cooper KD, Cordoro KM, Davis DM, Krol A, Margolis DJ, Paller AS, Schwarzenberger K, Silverman RA, Williams HC, Elmets CA, Block J, Harrod CG, Smith Begolka W, Sidbury R. Guidelines of care for the management of atopic dermatitis: section 1. Diagnosis and assessment of atopic dermatitis. Journal of the American Academy of Dermatology. 2014;70(2):338-51. Epub 2013/12/03. doi: 10.1016/j.jaad.2013.10.010. PubMed PMID: 24290431; PMCID: PMC4410183.
121. Olsen EA, Hordinsky MK, Price VH, Roberts JL, Shapiro J, Canfield D, Duvic M, King LE, Jr., McMichael AJ, Randall VA, Turner ML, Sperling L, Whiting DA, Norris D, National Alopecia Areata F. Alopecia areata investigational assessment guidelines--Part II. National Alopecia Areata Foundation. Journal of the American Academy of Dermatology. 2004;51(3):440-7. Epub 2004/09/01. doi: 10.1016/j.jaad.2003.09.032. PubMed PMID: 15337988.
122. Sartorius K, Killasli H, Heilborn J, Jemec GB, Lapins J, Emtestam L. Interobserver variability of clinical scores in hidradenitis suppurativa is low. Br J Dermatol. 2010;162(6):1261-8. Epub 2010/02/27. doi: 10.1111/j.1365-2133.2010.09715.x. PubMed PMID: 20184581.
123. Puzenat E, Bronsard V, Prey S, Gourraud PA, Aractingi S, Bagot M, Cribier B, Joly P, Jullien D, Le Maitre M, Paul C, Richard-Lallemand MA, Ortonne JP, Aubin F. What are the best outcome measures for assessing plaque psoriasis severity? A systematic review of the literature. J Eur Acad Dermatol Venereol. 2010;24 Suppl 2:10-6. Epub 2010/05/15. doi: 10.1111/j.1468-3083.2009.03562.x. PubMed PMID: 20443995.
124. Feily A. Vitiligo Extent Tensity Index (VETI) score: a new definition, assessment and treatment evaluation criteria in vitiligo. Dermatol Pract Concept. 2014;4(4):81-4. Epub 2014/11/15. doi: 10.5826/dpc.0404a18. PubMed PMID: 25396094; PMCID: PMC4230268.
125. Esbensen AJ, Hooper SR, Fidler D, Hartley SL, Edgin J, d'Ardhuy XL, Capone G, Conners FA, Mervis CB, Abbeduto L, Rafii M, Krinsky-McHale SJ, Urv T, Outcome Measures Working G. Outcome Measures for Clinical Trials in Down Syndrome. Ajidd-American Journal on Intellectual and Developmental Disabilities. 2017;122(3):247-81. doi: 10.1352/1944-7558-122.3.247. PubMed PMID: WOS:000404294100004.
126. Sahakian BJ, Morris RG, Evenden JL, Heald A, Levy R, Philpot M, Robbins TW. A comparative study of visuospatial memory and learning in Alzheimer-type dementia and Parkinson's disease. Brain. 1988;111 (Pt 3):695-718. Epub 1988/06/01. PubMed PMID: 3382917.
127. Howell CR, Gross HE, Reeve BB, DeWalt DA, Huang IC. Known-groups validity of the Patient-Reported Outcomes Measurement Information System (PROMIS((R))) in adolescents and young adults with special healthcare needs. Quality of life research : an international journal of quality of life aspects of treatment, care and rehabilitation. 2016;25(7):1815-23. Epub 2016/02/14. doi: 10.1007/s11136-016-1237-2. PubMed PMID: 26872912; PMCID: PMC4893933.
128. Cella D, Riley W, Stone A, Rothrock N, Reeve B, Yount S, Amtmann D, Bode R, Buysse D, Choi S, Cook K, Devellis R, DeWalt D, Fries JF, Gershon R, Hahn EA, Lai JS, Pilkonis P, Revicki D, Rose M, Weinfurt K, Hays R. The Patient-Reported Outcomes Measurement Information System (PROMIS) developed and tested its first wave of adult self-reported health outcome item banks: 2005-2008. Journal of clinical epidemiology. 2010;63(11):1179-94. Epub 2010/08/06. doi: 10.1016/j.jclinepi.2010.04.011. PubMed PMID: 20685078; PMCID: PMC2965562.
129. Ram G, Chinen J. Infections and immunodeficiency in Down syndrome. Clin Exp Immunol. 2011;164(1):9-16. doi: 10.1111/j.1365-2249.2011.04335.x. PubMed PMID: 21352207; PMCID: PMC3074212.
130. So SA, Urbano RC, Hodapp RM. Hospitalizations of infants and young children with Down syndrome: evidence from inpatient person-records from a statewide administrative database. J Intellect Disabil Res. 2007;51(Pt 12):1030-8. Epub 2007/11/10. doi: 10.1111/j.1365-2788.2007.01013.x. PubMed PMID: 17991010.
131. Perez-Padilla R, Fernandez R, Garcia-Sancho C, Franco-Marina F, Aburto O, Lopez-Gatell H, Bojorquez I. Pandemic (H1N1) 2009 virus and Down syndrome patients. Emerg Infect Dis. 2010;16(8):1312-4. Epub 2010/08/04. doi: 10.3201/eid1608.091931. PubMed PMID: 20678334; PMCID: PMC3298326.

132. Beckhaus AA, Castro-Rodriguez JA. Down Syndrome and the Risk of Severe RSV Infection: A Meta-analysis. Pediatrics. 2018;142(3). Epub 2018/08/11. doi: 10.1542/peds.2018-0225. PubMed PMID: 30093540.
133. van der Sluijs KF, van Elden LJ, Nijhuis M, Schuurman R, Pater JM, Florquin S, Goldman M, Jansen HM, Lutter R, van der Poll T. IL-10 is an important mediator of the enhanced susceptibility to pneumococcal pneumonia after influenza infection. J Immunol. 2004;172(12):7603-9. Epub 2004/06/10. PubMed PMID: 15187140.
134. Ballinger MN, Standiford TJ. Postinfluenza bacterial pneumonia: host defenses gone awry. Journal of interferon & cytokine research : the official journal of the International Society for Interferon and Cytokine Research. 2010;30(9):643-52. Epub 2010/08/24. doi: 10.1089/jir.2010.0049. PubMed PMID: 20726789; PMCID: PMC4367524.
135. Shahangian A, Chow EK, Tian X, Kang JR, Ghaffari A, Liu SY, Belperio JA, Cheng G, Deng JC. Type I IFNs mediate development of postinfluenza bacterial pneumonia in mice. J Clin Invest. 2009;119(7):1910-20. Epub 2009/06/03. doi: 10.1172/JCI35412. PubMed PMID: 19487810; PMCID: PMC2701856.
136. Lisnevskaia L, Murphy G, Isenberg D. Systemic lupus erythematosus. Lancet. 2014;384(9957):1878-88. Epub 2014/06/03. doi: 10.1016/S0140-6736(14)60128-8. PubMed PMID: 24881804.
137. Medicine USNLo. Studies for “Tofacitinib” and “Lupus” on ClinicalTrials.gov 2019 [04/03/2019]. Available from: https://clinicaltrials.gov/ct2/results?term=Tofacitinib&cond=lupus&rank=3#rowId2.
138. Eliot M, Ferguson J, Reilly MP, Foulkes AS. Ridge regression for longitudinal biomarker data. Int J Biostat. 2011;7(1):Article 37. Epub 2011/11/04. doi: 10.2202/1557-4679.1353. PubMed PMID: 22049265; PMCID: PMC3202941.
139. Sela RJ, Simonoff JS. RE-EM trees: a data mining approach for longitudinal and clustered data. Mach Learn. 2012;86(2):169-207. doi: 10.1007/s10994-011-5258-3. PubMed PMID: WOS:000300589300001.
